# Bit Allocation in Spatially Correlated Sensor Fields: A Comparative Study of Contribution-Aware and Heuristic Approaches

**DOI:** 10.3390/s26134265

**Published:** 2026-07-04

**Authors:** Sang-Seon Byun

**Affiliations:** Department of Computer Engineering, Catholic University of Pusan, Busan 46252, Republic of Korea; ssbyun@cup.ac.kr; Tel.: +82-51-510-0651

**Keywords:** bit allocation problem, Shapley value, cooperative game theory, stratified random sampling, mutual information

## Abstract

Bit allocation is a core design problem in spatially correlated sensor fields under limited communication resources since per-sensor bit depth determines quantization fidelity and thus the quality of acquired information. In this paper, we investigate several regime-dependent bit allocation strategies and compare them under various deployment geometries, bit budgets, and performance metrics. We consider a per-reporting-round integer bit-allocation problem in which a total bit budget is distributed among sensors as nonnegative quantization bits, allowing zero-bit allocation to represent sensor silencing. To examine different allocation principles, we compare five strategies: Shapley-value-based contribution-aware allocation and four other heuristic approaches—uniform allocation, Voronoi-based geometry-aware allocation, greedy mutual information-driven allocation, and conditional variance-based allocation. We implement the contribution-aware allocation as a two-stage framework: a mutual information-based cooperative game first quantifies each sensor’s spatial redundancy-aware contribution using Shapley value, and the value is then mapped to integer bit allocations. To mitigate the intractability of this formulation in larger networks, we approximate Shapley values via Neyman stratified sampling. Numerical experiments on sampled random fields show that reconstruction performance is context-dependent: geometry-aware allocation often performs best under tight budgets, particularly on boundary and tail errors, while Shapley-value-based allocation yields the best performance in stringent small-scale fields and becomes competitive under high budgets for global and tail errors. Furthermore, mutual information and weighted posterior trace provide complementary rankings, highlighting trade-offs between information-centric objectives and reconstruction-error objectives under heterogeneous spatial redundancy. These results show trade-offs among allocation strategies in accordance with different regimes and performance metrics.

## 1. Introduction

Wireless sensor networks (WSNs) have been widely used to monitor spatial phenomena such as environmental and agricultural monitoring, structural health monitoring, smart-city sensing, and disaster-response sensing.

A fundamental constraint in WSNs is the limited communication resources—e.g., bandwidth or energy—available for delivering each sensor’s measurement to a sink or fusion center, which makes quantization and bit allocation a critical design issue. Many previous studies related to WSNs have paid attention to transmission cost that dominates sensors’ resource consumption and network design for managing constrained transmission resources while maintaining monitoring fidelity [1].

In many WSNs that monitor especially spatial phenomena, sensor measurements generally exhibit significant spatial correlation. This implies that allocating transmission resources such as quantization bits uniformly to all sensors often degrades measurement efficiency; densely located sensors may convey redundant information, while isolated ones may provide more distinct information about the field. Gaussian process (GP) models provide a principle for representing the feature of spatial correlation and for quantifying predictive uncertainty and information gain under partial observations [2]. Information-theoretic criteria—most notably mutual information (MI)—have been widely adopted in sensor placement (or selection) and resource allocation problems in spatially correlated WSNs since they can estimate the amount of information given by a set of sensors [3,4].

Generally, resources such as transmission power and bandwidth are finite in WSNs; therefore, they need to be allocated in a proper way. In this paper, we consider a per-reporting-round bit-allocation problem in spatially correlated sensor fields under a total bit-budget constraint. The total bit budget is distributed among sensors as nonnegative integer quantization bits, where a zero-bit allocation is allowed to represent sensor silencing. Furthermore, this available bit budget may vary over time depending on the transmission resources available in each reporting round. Therefore, the problem considered in this study is to determine how to allocate a limited bit budget across sensors while accounting for spatial redundancy, deployment geometry, and the target performance objective.

In spatially correlated sensor fields, the amount of exclusive information provided by each sensor is not independent of the other sensors; densely located sensors may provide redundant information, whereas isolated or sparsely located ones may provide more distinct information. Consequently, the effectiveness of a bit-allocation strategy depends on deployment geometry, correlation structure, total available bit budget, and performance metric of interest.

To this end, we introduce and compare five allocation strategies: Shapley-value-based contribution-aware allocation and four other heuristic approaches—uniform allocation, Voronoi-based geometry-aware allocation, greedy mutual information (MI)-driven allocation, and conditional variance-based allocation. These strategies represent distinct allocation principles, including coalitional contribution, equal sharing, geometric coverage responsibility, individual information gain, and conditional novelty.

We evaluate the allocation strategies using complementary information-theoretic, uncertainty-based, and reconstruction-error metrics, including MI, weighted posterior trace, global RMSE, boundary RMSE, and worst-10% RMSE. These metrics are not aggregated into a single scalar score because they represent different design objectives. Instead, conflicting rankings are treated as evidence of trade-offs among allocation principles.

Among the allocation strategies, the Shapley-value-based approach is implemented as a contribution-aware allocation strategy. In order to implement this approach in a principled manner, we propose a two-stage framework. In the first stage, an MI-based cooperative game is applied to quantify each sensor’s spatial redundancy-aware contribution using Shapley value. The Shapley value provides an axiomatic solution concept to score each sensor’s average marginal contribution across all possible sensor coalitions, thereby capturing redundancy among spatially correlated sensors [5]. In the second stage, the resulting scores are mapped to nonnegative integer bit allocations under a total bit budget constraint. This mapping provides an interpretable contribution-aware allocation rule. This two-stage framework—which measures each player’s contribution score to the collective payoff and subsequently allocates resources or costs in proportion to the score—is well established and widely applied in various cooperative games, such as voting games and airport games.

However, calculating the exact Shapley value is often computationally intractable since it requires evaluating each player’s marginal contributions over all possible coalitions, which hinders its real-world deployment [5]. To tackle this intractability, we approximate Shapley values using a Neyman-stratified sampling approach that leverages complementary contributions and optimal sample allocation rules to minimize estimator variance for a given sample budget [6,7]. This approach enables efficient Shapley-value-based bit allocation in WSNs where utility evaluations (e.g., MI under GP models) are themselves nontrivial.

The remainder of this paper is organized as follows. Section 2 briefly reviews related work in the context of resource allocation in WSNs. Section 3 details several system models employed throughout this study. Section 4 introduces the allocation strategies, including a Shapley-value-based bit-allocation framework. Section 5 details the experimental setup and evaluation protocols. Section 6 provides the comparative numerical results and discusses regime-dependent trade-offs among the allocation strategies. Finally, Section 7 concludes this paper.

## 2. Related Work

### 2.1. Communication Constraints and Resource-Efficient WSN Design

WSNs typically operate under stringent resource constraints, where wireless communication dominantly influences energy consumption and system lifetime. Therefore, this motivates network designs that can manage transmissions carefully. Recent surveys have emphasized communication-efficient operations (e.g., aggregation and clustering) as core design principles for enhancing the sustainability of sensor monitoring [8,9]. In this study, we focus on bit-budget constraints as an abstraction of limited bandwidth in periodic sensing and reporting.

### 2.2. Spatially Correlated Fields and Information-Theoretic Criteria (GP/MI)

Spatial phenomena often involve strong spatial correlation among the values to be measured. GPs provide a principled framework for modeling such correlated fields and for quantifying uncertainty under partial observations [2].

Information-theoretic criteria—especially MI—have been widely used to quantify the value of observations for learning or reconstructing latent fields. A few representative studies have addressed near-optimal sensor placement (or selection) within the framework of GP models by exploiting the structure of MI (e.g., submodularity) to enable efficient greedy approximations with provable guarantees [3].

In contrast to the sensor placement problem, our work focuses on the precision of data to be reported—that is, allocating a limited number of quantization bits across sensors. This allocation becomes particularly crucial when redundancy is inherent in both the spatial correlation and the deployment geometry.

### 2.3. Bit (Bit-Rate) Allocation Under Communication Constraints

Bit allocation (or bit-rate allocation) has been studied in various estimation or control domains, where quantization resolution directly impacts both bandwidth consumption and estimation accuracy. Recently, Ref. [10] has addressed bit-rate-constrained distributed filtering, explicitly considering the effects of protocol-level rate allocation on estimation accuracy in WSNs. The authors have proposed a channel coding scheme that filters error dynamics in sensor networks, aiming to maximize filtering performance subject to the bit rate constraint in sensor communication links.

A complementary study [11] has analyzed bit-depth allocation from an optimization perspective. By employing a relaxed bit-depth formulation, the authors have established convexity results for LMMSE (linear minimum mean square error) estimation problems, yielding globally optimal allocations subject to discretization.

The joint allocation of bits and power [12] has been investigated to maximize the transmission rate in sensor communications with MIMO (Multiple-Input–Multiple-Output) channels. However, this work has not considered spatial correlation among sensors.

In [13], the authors studied bit allocation for tracking moving targets using sensors under a fixed total bit budget. Tracking performance is defined in terms of the determinant of each sensor’s Fisher information matrix.

The problem of determining the minimum transmission bits [14] has been studied under constraints on fixed transmission energy consumption and tracking accuracy, assuming that all sensors transmit the same number of bits.

In [15], a rate-constrained bit-allocation problem in sensor networks was studied, taking into account multiple points of interest and non-uniform error requirements.

In [16], an information-based bit-allocation scheme was developed for cooperative visual sensing in vehicular networks with the goal of allocating quantization bits to minimize the overall sensing error. This work also employed the Fisher information matrix to evaluate sensing performance.

We briefly compare our work with a few prior studies [10,13,16] that have addressed rate or bit allocation in filtering, tracking, and cooperative visual sensing problems in Table 1, and it is found that none of these prior studies consider integer bit allocation for reconstructing GP-based spatially correlated random fields. In contrast, this work studies methods for reconstructing spatially correlated random fields by scoring sensor contributions through MI-based cooperative game and other heuristic baselines.

### 2.4. Geometry-Aware Allocation and MI-Greedy Information Gathering

Voronoi diagram is a canonical geometric tool for partitioning a sensing region into per-sensor responsibility cells based on geometric proximity. It has been widely adopted in coverage control and spatial task allocation. Early coverage-control formulations explicitly define sensing utilities based on Voronoi tessellations to achieve distributed and interpretable geometry-aware strategies [7,16].

In the context of WSNs, Voronoi partitions facilitate the distributed measurements of cells and drive geometry-aware decisions—most notably for field coverage and sensor clustering. It often serves as a lightweight baseline that reflects geometric coverage responsibility [17].

More recently, weighted Voronoi tessellations have been applied to account for heterogeneous sensing capabilities, enabling the design of coverage strategies that explicitly encode spatial responsibility in such diverse sensing conditions [18].

A Voronoi diagram itself is not an information-aware sensing model since it does not use covariance, MI, posterior uncertainty, measurement noise, or redundancy among sensor observations. Nonetheless, we include the allocation based on the Voronoi diagram since it provides a geometry-aware heuristic baseline that tests whether spatial coverage alone can guide bit allocation effectively. That is, in sensor fields, the amount of information associated with a sensor may be related to the area of the region monitored by that sensor even though this approach does not reflect information redundancy caused by spatial correlation. Consequently, it is used as a computationally simple coverage proxy against which MI-driven, conditional variance-based, and contribution-aware allocation strategies can be compared.

The greedy approach under the MI objective is widely used due to its reasonable computational complexity and effective exploitation of spatial correlation. Generally, MI exhibits submodularity in stationary sensor fields, which allows for greedy selection with provable near-optimal bounds and strong empirical performance for sensor selection and placement [3]. Consequently, greedy MI-driven approaches serve as a natural baseline by exploiting spatial correlation of sensor fields and prioritizing observations expected to reduce field uncertainty. However, they typically optimize a set-selection (or sequential acquisition) objective rather than an explicit bit-budgeted quantization allocation across all sensors. This distinction motivates our comparison between contribution-aware Shapley-value-based bit allocation and greedy MI-driven allocations under identical bit-budget constraints.

### 2.5. Practical Sensor Network Applications

Recent sensor network studies show that practical WSN and IoT systems often require resource-aware operation under application-specific constraints. For example, emergency rescue networks based on air-to-ground integrated mobile ad hoc networks face dynamic topology, limited node resources, and unstable communication opportunities [19]. Such scenarios require adaptive communication decisions that consider node position, communication reliability, and resource availability. Although this line of work focuses on routing and message delivery rather than bit allocation, it illustrates the importance of resource-aware communication in mission-critical sensor network environments.

Smart city sensing provides another relevant example. In heterogeneous WSNs for smart city IoT systems, physically adjacent sensors can generate sensor data with similar information, and this spatial similarity can be exploited for collaborative data validation and anomaly detection [20]. This observation is closely related to the motivation of the present work: when neighboring sensors provide redundant or similar information, reporting all measurements with the same quantization resolution may be inefficient.

These practical studies motivate the need for allocation mechanisms that account for both resource constraints and spatial relationships among sensor observations. In this work, we address this complementary problem by allocating a fixed reporting bit budget among spatially correlated sensors for field reconstruction. The proposed framework is therefore relevant to practical WSN applications where sensor measurements are spatially correlated and reporting resources are limited, such as environmental monitoring, smart-city sensing, structural health monitoring, and disaster-response sensing.

### 2.6. Summary of Gaps and Positioning

Prior work has established several important foundations for resource allocation in WSNs: (i) communication and energy constraints in WSN design [11,12], (ii) practical resource-aware applications [19,20], (iii) GP/MI-based sensing and greedy acquisition [2,3], (iv) bit- or rate-constrained estimation and control formulations [10,11], and (v) contribution-aware payoff allocation with the scalable Shapley value approximation method [21]. However, several gaps remain in applying these foundations to the bit-allocation problem in spatially correlated sensor fields.

First, bit-budgeted measurement reporting in spatially correlated fields has not been sufficiently examined under nonnegative integer bit constraints. In particular, the problem involves both bit-depth assignment and implicit sensor silencing, which make the allocation problem different from continuous rate allocation or conventional sensor selection. Second, existing allocation methods often rely on a single allocation principle, such as equal sharing, geometric coverage, information gain, or local uncertainty, without systematically comparing how different principles behave under various deployment geometries and bit budget regimes. Third, the relationship between information-centric metrics and reconstruction-error metrics remains insufficiently clarified, even though these metrics may rank allocation strategies differently.

These gaps lead to several technical challenges. The first challenge is to obtain a per-sensor contribution score that accounts for spatial redundancy since the usefulness of information that a sensor has depends on what the other sensors already have. The second challenge is to evaluate allocation strategies under multiple metrics without reducing them to an arbitrary single weighted score since MI, global reconstruction error, boundary error, tail error, and posterior uncertainty capture different design objectives. Finally, when Shapley values are used, exact computation becomes infeasible as the number of sensors increases, requiring a scalable approximation method.

This paper addresses these gaps by comparing five allocation strategies under a unified experimental framework: Shapley-value-based allocation, uniform allocation, Voronoi-based allocation, greedy MI-driven allocation, and conditional variance-based allocation. The Shapley-value-based method is included as a contribution-aware strategy that captures average marginal contributions over sensor coalitions. The other methods represent equal-sharing, geometry-aware, information-driven, and variance-based allocation principles. Then our main goal is to identify which allocation strategy is more effective under different deployment geometries, bit-budget regimes, and performance metrics.

Furthermore, the two-stage approach—utilizing Shapley value primarily as an inherent contribution score to guide subsequent resource or cost distribution—is well-established and highly intuitive in many cooperative games. Historically, a wide range of resource or cost allocation problems have successfully decoupled the measurement of marginal contributions from the physical constraints of the final assets. Classical examples include voting games, where the Shapley value measures abstract voting power [22], and airport games, where it quantifies the fundamental cost responsibilities used to derive specific aircraft landing fees [23,24]. Hereby, the Shapley value in our two-stage framework is a principled redundancy-aware contribution metric that serves as a solid foundation for the final integer bit allocation.

Fisher information-based methods can also be employed for our problem. However, they are not directly aligned with the integer bit-allocation problem for reconstructing spatially correlated random fields without additional assumptions such as a specific parameterization of the sensor fields, a particular information-matrix objective, or a separate rule for converting the resulting matrix criterion into per-sensor bit allocations. On the other hand, the MI-based cooperative game formulation directly yields redundancy-aware contribution scores of sensors, and these scores can be directly mapped to bit allocations. Therefore, in this work, we focus on an MI-based cooperative game formulation while leaving Fisher information-based bit allocation as a candidate for our future study.

Beyond sensor-network-specific studies, broader allocation and resource-distribution problems have also been investigated in spatially distributed systems. For example, recent location-allocation studies have considered joint facility-location, allocation, and routing decisions under multiple competing objectives and heuristic solution strategies [25]. Other work has examined spatial equality and equity in multiperiod allocation problems for emergency resource distribution [26]. Although these studies address different application domains, they highlight a common methodological issue: limited resources must be allocated across spatially distributed entities while balancing competing performance objectives. The present work differs by focusing on quantization-bit allocation in spatially correlated sensor fields, but it shares the broader concern of comparing alternative allocation principles under multiple evaluation criteria.

Hereafter, we abbreviate “contribution-aware Shapley-value-based allocation” as “Shapley allocation”, “geometry-aware Voronoi allocation” as “Voronoi allocation”, “greedy MI-driven allocation” as “greedy allocation”, and “conditional variance-based allocation” as “CVWA”.

## 3. System Model and Problem Formulation

This section presents the sensor field model, the bit-budgeted quantization model, and the contribution-scoring framework used throughout the paper.

### 3.1. Spatial Field Model

Consider a two-dimensional sensing region discretized into a set of grid locations:

(1) U={1,2,…,|U|},

where the field value at grid point *u* ∈ *U* is denoted *X_u_*. All grid values are given by the vector

(2) $$X_{U}\in R^{\left|U\right|}.$$

We assume a zero-mean GP prior over the discretized field:

(3) $$X_{U}\sim N\left(0,K_{UU}\right),$$

where **K***_UU_* is a covariance matrix generated by an isotropic covariance model given in [3]. We let γ=α‖i−j‖ for α>0. Then the correlation function is

(4) $$\rho \left(i,j\right)=\frac{1}{\pi }\left(\frac{\left(2\pi -\gamma \right)\left(1+\cos(\gamma /2)\right)}{3}+\frac{1}{2}\sin\gamma \right),$$

if γ<2π and zero otherwise. For two grid points *i* and *j*, the covariance *K*(*i*, *j*) = *σ*_f_^2^*ρ*(*i*, *j*) where *σ*_f_^2^ denotes the field variance scale. In our experiments, we let *σ*_f_^2^ = 1.

Let *S*⊂*U* be the set of grid indices where sensors are placed with |*S*| = *N*. The sensor measurement vector is

(5) $$X_{S}=\left\{X_{u}:u\in S\right\}.$$

We denote the complement grid set by $\bar{S}=U\backslash S.$

### 3.2. Bit-Budgeted Quantization Model

Each sensor *i* ∈ *S* is assigned an integer number of quantization bits *b_i_* (≥0). A total bit-budget constraint is given by

(6) $$\sum_{\forall i\in S}b_{i}\leq B.$$

where *B* denotes the total bit budget and is interpreted as a per-reporting-round communication budget. We also allow *b_i_* = 0, which represents silencing (no transmission) under stringent budgets.

The bit allocation considered in this paper is a pre-measurement allocation problem. That is, the allocation vector **b** is determined before the actual field realization, sensor measurements, and quantization errors are observed. Therefore, the allocation strategies cannot reflect actual measurement noise or quantization error when assigning bits. Instead, each strategy determines the bit allocation using prior information available before measurement reporting, such as sensor deployment geometry, correlation structure, MI-based contribution, or conditional variance. After the allocation vector is fixed, sensor measurements are quantized according to the assigned bit depths, and the resulting quantization effect is reflected in the reconstruction and evaluation stages.

To incorporate quantization effects analytically, we adopt a standard additive quantization-noise approximation:

(7) $${\tilde{X}}_{i}=X_{i}+Q_{i},$$

where *Q_i_* is independent of *X_i_* and modeled as zero-mean with variance representing exponential decay with bits

(8) $$\sigma_{q,i}^{2}\left(b_{i}\right)=k_{i}2^{-2b_{i}},$$

where *k_i_* is the signal variance at sensor *i*, that is, *k_i_* = Var(*X_i_*). This model captures the key trade-off: increasing *b_i_* reduces quantization distortion at the cost of consuming the bit budget.

Let ${\tilde{X}}_{S}$ be the quantized observation vector and define the diagonal quantization-noise covariance

(9) $$\Sigma_{q}\left(b\right)={\mathrm{diag}\left(\sigma_{q,i}^{2}\left(b_{i}\right)\right)}_{i\in S}.$$

Then, conditioned on **X***_S_*, the quantized observation model is

(10) $${\tilde{X}}_{S}|X_{S}\sim N\left(X_{S},\Sigma_{q}\left(b\right)\right).$$

### 3.3. GP Reconstruction with Bit-Dependent Observation Quality

Given ${\tilde{X}}_{S}$ and the GP prior, the posterior distribution of the full field **X***_U_* remains Gaussian. We denote the prior covariance blocks for a set of sensors *S*: **K***_US_*, **K***_SU_*, **K***_SS_*, and **K***_UU_*. Then the posterior mean used for field reconstruction is

(11) $$\mu_{U|\tilde{S}}=K_{US}{\left(K_{SS}+\Sigma_{q}\left(b\right)\right)}^{-1}{\tilde{X}}_{S},$$

and the posterior covariance is

(12) $$K_{U|\tilde{S}}=K_{UU}-K_{US}{\left(K_{SS}+\Sigma_{q}\left(b\right)\right)}^{-1}K_{SU},$$

where **Σ***_q_*(**b**) = diag(σ^2^*_q_*_,*i*_(*b_i_*)) and σ^2^*_q_*_,*i*_(*b_i_*) = $k_{i}2^{-2b_{i}}$. *k_i_* is a constant that scales the variance of quantization error, and we let *k_i_* = [**K***_SS_*]*_ii_*.

These formulations show how a given bit-allocation vector affects reconstruction quality through bit-dependent observation noise. Allocating more bits to one sensor reduces its quantization noise, but the resulting reconstruction benefit also depends on the measurements and bit allocations of other sensors due to spatial correlation. In this study, the bit-dependent model is used for reconstruction and performance evaluation after each allocation strategy has produced an allocation vector. Developing a bit-allocation strategy that optimizes a joint bit-dependent reconstruction utility is left as future work.

### 3.4. Information-Theoretic Utility of a Sensor Coalition

To quantify the information contribution of a subset of sensors, we use MI. In our formulation, a coalition corresponds to selecting a subset of sensor locations. Let *C* ⊆ *S* be a sensor coalition, and define its complement in the grid as $\bar{C}=U\backslash C$. Then we define the utility provided by *C* as

(13) $$v\left(C\right)≜I\left(X_{C};X_{\bar{C}}\right).$$

Under the Gaussian prior, MI yields a closed form in terms of log-determinants as

(14) $$I\left(X_{C};X_{\bar{C}}\right)=\frac{1}{2}\log\frac{\left|K_{CC}\right|\left|K_{\bar{C}\bar{C}}\right|}{\left|K_{UU}\right|}.$$

This utility yields a larger value with coalitions that naturally provide more exclusive information about the areas where no sensor is located, while capturing redundancy: adding a new sensor near an already-saturated area yields a diminishing marginal gain.

In the game definition that is to be described in the next subsection, *v*(*C*) is independent of bit allocation: instead, bit allocation is performed after quantifying each sensor’s contribution using the Shapley value obtained from this MI-based cooperative game.

We also note that *v*(*C*) should be distinguished from the bit-dependent MI metric given in (31) for performance evaluation. The utility *v*(*C*) is a pre-allocation, bit-independent measure for computing Shapley-value-based contribution scores, whereas the bit-dependent MI metric is evaluated after the bit-allocation vector is determined and includes quantization noise effects. Therefore, both the *v*(*C*) and MI metrics use information-theoretic concepts but they serve different roles in our framework.

### 3.5. Cooperative Game Theoretic Formulation

We define a cooperative game (*S*, *v*), referred to as a bit-allocation game, where the players are sensors (denoted *S*), and the coalition payoff *v*(*C*) is given by (13).

Crucially, the coalition payoff *v*(*C*) is inherently bit-independent, serving exclusively to quantify each sensor’s spatial redundancy-aware contribution before any bit allocation is performed. Consequently, our formulation for the contribution-aware allocation is designed as a two-stage framework rather than a fully bit-dependent cooperative game.

The purpose of the cooperative game-theoretic formulation is to quantify a per-sensor contribution score *Ø_i_*, that is, a Shapley value, that satisfies the standard fairness axioms (symmetry, dummy player, additivity, efficiency) [27]. For each sensor *i*, its Shapley value is defined as

(15) $$\emptyset_{i}=\sum_{C\subseteq S-\left\{i\right\}}\frac{\left(N-\left|C\right|-1\right)!\left|C\right|!}{N!}\times {∆}_{i}v\left(C\right)$$

where ${∆}_{i}v\left(C\right)=v\left(C\cup \left\{i\right\}\right)-v\left(C\right).$ Intuitively, *Ø_i_* represents the expected marginal contribution of sensor *i* averaged over all possible joining orders.

The Shapley value functions as a metric for sensor contribution; a higher value indicates that the sensor provides more exclusive information by effectively mitigating spatial redundancy. These resulting contribution scores are then mapped to specific integer bit allocations within the total available bit budget.

A fully bit-dependent game *v*(*C*, **b***_C_*) would jointly model coalition membership and allocated bit depths, but this would require a substantially complicated joint optimization over sensor subsets and bit allocations.

### 3.6. Bit Allocation via Shapley Value

Given contribution scores {*Ø_i_*}*_i_*_∈*S*_, we allocate the total bit budget *B* in proportion to the scores as follows:

(16) $$b_{i}^{*}\propto \max\left({Ø}_{i},0\right),\;\sum_{i}b_{i}\leq B,\;b_{i}^{*}\in Z_{\geq 0}.$$

This is followed by integer rounding and the redistribution of any remaining bits. Allowing *b_i_* = 0 enables sensor silencing when *Ø_i_* is negligible. Specifically, we apply floor rounding (round-down) and allocate the remaining bits to the sensors with the higher contribution scores according to each respective allocation method: Shapley value, MI gained individually, Voronoi coverage area, and conditional variance.

This proportional mapping is adopted as a simple and interpretable mechanism for converting contribution scores into integer bit allocations. We do not claim that this mapping is globally optimal with respect to quantization-noise-aware reconstruction objectives. Instead, it defines one contribution-aware allocation strategy that can be compared with geometry-aware, information-driven, and variance-based alternatives. Accordingly, (16) should be interpreted as a contribution-to-bit mapping rule, not as a globally optimal solution to a bit-dependent reconstruction objective.

The use of Shapley values for bit allocation follows a common interpretation in cooperative game theory: after a coalition game is defined, each player’s Shapley value can be used as a contribution-based share for allocating payoff, cost, power, or responsibility. Classical examples include voting games and airport cost-sharing games. In the present problem, the “players” are sensors, the coalition value is the MI-based information utility, and the Shapley value is used as a spatial redundancy-aware contribution score for subsequent bit allocation.

### 3.7. Computational Challenge and Sampling-Based Approximation

Direct computation of the Shapley value is combinatorial, requiring evaluations across 2*^N^* subcoalitions. Consequently, the exact Shapley value becomes computationally intractable even for moderate *N* [5]. In the next section, we present an approximation approach based on stratified random sampling, referred to as the Neyman approach as well.

This sampling-based approximation reduces the computational burden dramatically compared with exact Shapley value computation and enables contribution-aware allocation for moderate-size networks. However, it still takes tens of minutes—as discussed in Section 6.2—to obtain approximate Shapley values even for *N* = 40, which prevents them from being deployed in fully scalable real-time solutions for arbitrarily large WSNs. Therefore, this approximation is best viewed as a tractability improvement over exact Shapley value computation, rather than as a complete solution to very large-scale scalability.

## 4. Bit-Allocation Strategies

This section presents the bit-allocation strategies. We first describe the contribution-aware Shapley allocation scheme, and then give the baselines of the other strategies, including their implications.

### 4.1. Shapley Allocation

#### 4.1.1. Overview of Two-Stage Framework

Given a set of sensors *S* (|*S*| = *N*) and the GP prior over the discretized field **X***_U_*, the allocation pipeline consists of

1. **Contribution scoring**, where Shapley value *Ø_i_* is computed for each sensor *i* ∈ *S* in the cooperative game (*S*, *v*) with $v\left(C\right)=I\left(X_{C};X_{\bar{C}}\right);$

2. **Bit mapping**, which converts *Ø_i_* into nonnegative integer bit allocations **b** = (*b_i_*)*_i_*_∈*S*_ subject to the constraint Σ*_i_b_i_* ≤ *B*, permitting *b_i_* = 0;

#### 4.1.2. Computation of Exact Shapley Values

For sensor *i*, the Shapley value is defined as the average marginal contribution across all possible joining orders, using the utility function *v*() from (13) and (14):

(17) $$\emptyset_{i}=\frac{1}{N!}\sum_{\pi \subseteq \prod \left(S\right)}\left[v\left({\mathrm{Pre}}_{i}\left(\pi \right)\cup \left\{i\right\}\right)-v\left({\mathrm{Pre}}_{i}\left(\pi \right)\right)\right]$$

where ∏(S) is the set of all permutations and Pre*_i_*(*π*) denotes the set of sensors preceding *i* in permutation *π*. An equivalent subset-based formulation is provided in (15).

Each marginal contribution requires multiple log-determinant evaluations of the submatrices of the covariance **K***_UU_*. Consequently, the exact computation scales exponentially with *N*, rendering it computationally prohibitive even for moderate sensor counts.

#### 4.1.3. Approximation via Stratified Random Sampling

To mitigate the computational complexity of the exact Shapley value, we adopt a coalition-size stratification, where subsets are grouped according to their cardinality [6].

For sensor *i*, the coalition-size stratum *k* ∈ {0, 1, …, *N* − 1} is defined as *C* ⊆ *S*\{*i*} with |*C*| = *k*. We denote the set of all possible coalitions in this stratum as *C_ik_*; then, |*C_ik_*| = $\left(\begin{array}{c} N-1\\ k \end{array}\right)$. Thus, the number of possible coalitions varies significantly with *k* and grows combinatorially with *N*, especially for middle coalition size. This combinatorial growth is one of the main reasons exact Shapley value computation becomes infeasible for large sensor networks.

We define the marginal contribution of sensor *i* as

(18) $${∆}_{i}^{\left(k\right)}\left(C\right)≜v\left(C\cup \left\{i\right\}\right)-v\left(C\right).$$

The mean marginal contribution of sensor *i* within stratum *k* is then

(19) $$\mu_{i,k}=\frac{1}{\left(\begin{array}{c} N-1\\ k \end{array}\right)}\sum_{{C\in C}_{ik}}{∆}_{i}^{\left(k\right)}\left(C\right).$$

Using this stratum mean, the Shapley value is rewritten as

(20) $$\emptyset_{i}=\frac{1}{N}\sum_{k=0}^{N-1}\mu_{i,k}.$$

This decomposition shows that the coalition count |*C_ik_*| is used to define the stratum mean. After the Shapley weighting is applied, however, each coalition-size stratum contributes equally with weight 1/*N*. Therefore, the approximation estimates *μ_i_*_,*k*_ independently for each *k* using uniformly sampled coalitions from *C_ik_*, and then averages the estimated stratum means.

#### 4.1.4. Neyman Approach for Optimal Sample Distribution

Let *m_k_* be the number of samples assigned to stratum *k* subject to the total sampling budget *M*. For each *k*, let *σ*^2^*_i_*_,*k*_ denote the variance of Δ^(*k*)^*_i_*(*C*) when *C* is sampled uniformly from *C_ik_*. In practice, *σ*^2^*_i_*_,*k*_ is estimated using a small number of pilot samples [6].

Following the mixed integer nonlinear programming (MINLP) proposed in [22], we determine *m_k_* by minimizing the aggregate estimated variance of the Shapley value estimators. In particular, Ref. [22] exploits the complementary-contribution with pairing strata *k* and *N*-*k*. Accordingly, *m_k_* is obtained by solving the following MINLP:

(21) $$\mathrm{Minimize}\;\frac{1}{N}\sum_{k=\left\lceil N/2\right\rceil }^{N}\frac{\sum_{i=1}^{N}\frac{\sigma_{i,k}^{2}}{k}+\frac{\sigma_{i,n-k}^{2}}{n-k}}{m_{k}}$$

(22) $$\mathrm{subject}\;\mathrm{to}\;\sum_{k=\left\lceil N/2\right\rceil }^{N-1}m_{k}=M,$$

(23) $$1\leq m_{k}\leq \left(\begin{array}{c} N-1\\ k \end{array}\right),for\;k=\left\lceil N/2\right\rceil ,\;\ldots ,\;N-1.$$

The constraint in (23) indicates that the allocated sample size is bounded by $\left(\begin{array}{c} N-1\\ k \end{array}\right)$, assuming sampling without replacement. Then, the Neyman approach assigns more samples to strata with larger intra-stratum variance of marginal contributions, rather than simply assigning more samples to strata containing more possible coalitions.

#### 4.1.5. Detailed Procedure of the Approximation

Here we present the approximation procedure step-by-step.

1. For each sensor *i*, divide coalitions *C* ⊆ *S*\{*i*} into coalition-size strata according to |*C*| = *k*.
2. Draw pilot samples uniformly from each nonempty stratum and estimate the intra-stratum variance of the marginal contribution.
3. Allocate the total sampling budget across strata using the MINLP defined in (21)~(23).
4. For each stratum, uniformly sample the assigned number of coalitions without replacement, bounded by the number of available coalitions.
5. Compute the marginal contribution for each sampled coalition.
6. Estimate the mean marginal contribution in each stratum and combine the stratum-wise estimates to obtain the approximate Shapley value.

#### 4.1.6. Allocation Procedure

Given Shapley value *Ø_i_*, we convert them into nonnegative allocation weights for bit assignment. Since the number of allocated bits should be nonnegative, negative contribution scores cannot be directly used as bit-allocation weights. Therefore, we define

(24) $${\tilde{\emptyset }}_{i}={\left[\emptyset_{i}\right]}_{+}=\max\left(\emptyset_{i},0\right).$$

We then allocate bits in proportion to these weights as follows:

(25) $${\bar{b}}_{i}=B\frac{{\tilde{\emptyset }}_{i}}{\sum_{j\in S}{\tilde{\emptyset }}_{j}}.$$

Floor rounding is applied to the real-valued ${\bar{b}}_{i}$, after which any residual bits are distributed to sensors in descending order of their Shapley values. That is,

1. Set *b_i_* ← $\left\lfloor {\bar{b}}_{i}\right\rfloor$.

2. Distribute (*B* − Σ*_i_b_i_*) bits one-by-one to sensors with the highest ${\tilde{\emptyset }}_{i}$ until *B* is exhausted.

Since *b_i_* = 0 is allowed, sensors with small ${\tilde{\emptyset }}_{i}$ may be silenced under tight budgets.

### 4.2. Heuristic-Based Allocation Strategies

The four heuristic-based approaches are selected to represent distinct allocation principles. Uniform allocation represents the equal sharing principle and provides a simple non-adaptive reference. Voronoi allocation represents the geometry-aware coverage principle by assigning more bits to sensors covering larger spatial regions. Greedy allocation represents the individual information-gain principle by sequentially assigning bits according to incremental mutual information. CVWA represents the uncertainty-aware principle by prioritizing sensors with larger conditional variance after accounting for spatial correlation.

- Uniform allocation
  a. Equal sharing without spatial or statistical information.
  b. *b_i_* = ⌊B⁄N⌋ with the remainder distributed uniformly among randomly selected sensors.

- Voronoi allocation
  a. Geometry-aware coverage responsibility.
  b. Bits are allocated in proportion to the area of each sensor’s Voronoi cells (reflecting lower local density):
    (26) $${\bar{b}}_{i}=B\frac{Area(i)}{\sum_{i\in S}Area\left(i\right)}.$$
    where *Area*(*i*) denotes the size of the Voronoi cells assigned to sensor *i*. Any residual bits are distributed one-by-one to sensors in descending order of their cell areas.
  c. This method is not information-aware in the sense of using covariance, MI, posterior uncertainty, quantization noise, or redundancy among sensor measurements. Its role is to evaluate how effective this simple spatial-coverage proxy is compared with statistically or information-theoretically motivated allocation strategies.

- Greedy allocation
  a. Information-gain-based prioritization.
  b. Bits are allocated in proportion to individual MI gains:
    (27) $${\bar{b}}_{i}=B\frac{v(\{i\})}{\sum_{i\in S}v\left(\left\{i\right\}\right)}.$$

- CVWA
  a. Allocation in proportion to the uncertainty of each sensor.
  b. Bits are allocated in proportion to a correlation-aware novelty score Var(*X_i_* | **X***_S_*_\{*i*}_), which favors sensors that remain highly uncertain given the observations of others.
  c. Consistent with the other heuristics, residual bits are distributed one-by-one to sensors in descending order of their novelty scores.

### 4.3. Complexity

As discussed in Section 3, computing the exact Shapley value is prohibitive as it requires evaluating coalitional utilities across all possible subsets. In the subset formulation, this results in a computational complexity of O(2*^N^*) utility evaluations per sensor. The stratified sampling approximation requires O(*M*) sampled marginal contribution evaluations per sensor, and, consequently, it requires O(*NM*) evaluations for all sensors in the worst case. Additionally, the pilot stage for estimating *σ*^2^*_i_*_,*j*_ requires *O*(*NM*) evaluations as these variances should be estimated for each sensor and stratum.

Although the sampling-based approximation changes the exponential dependence of exact Shapley value computation into a sample budget-dependent procedure, the resulting cost can still be high. In the present implementation, each sampled marginal contribution requires evaluating coalition utilities based on covariance submatrices and log-determinants. Therefore, the practical runtime depends not only on *N* and *M* but also on the cost of each GP-based MI evaluation. If we let *M* increase in proportion to *N* to maintain approximation quality, the wall clock runtime may grow substantially for very large networks. Thus, the current implementation is suitable mainly for moderate-size networks or offline allocation settings. Scaling the method to hundreds or thousands of sensors would require additional approximations, such as Owen values [28], sparse GP approximations, or parallel computation of marginal contributions.

## 5. Experimental Setup and Evaluation Protocol

This section describes the experimental setup used to evaluate bit-allocation strategies in stationary, spatially correlated sensor fields. We detail (i) the field and sensor deployment configurations; (ii) the bit budget settings and allocation methods; (iii) the reconstruction procedure; (iv) the performance metrics; and (v) the evaluation scenarios.

### 5.1. Field Model and Data Generation

We consider a two-dimensional sensing region discretized into a uniform grid of 50 × 50, yielding |*U*| = 2500 grid points. The latent field **X***_U_* is drawn from the stationary GP prior given in (3) and (4). When constructing **K***_UU_* under finite-precision arithmetic, slight indefiniteness may occur. To ensure valid sampling and consistent GP inference, we project the covariance matrix onto the positive semidefinite cone via eigenvalue clipping:

(28) $$K\leftarrow Q\;\max\left(\Lambda ,\epsilon I\right)Q^{T}$$

where **K** = **QΛQ***^T^* is the eigen-decomposition and ϵ>0 is a small threshold (e.g., 10^−10^). The projected covariance is used consistently for (i) field sampling, (ii) posterior reconstruction, and (iii) the computation of covariance-based metrics.

For each experimental condition—encompassing deployment type, bit budget, and allocation method—we generate *N_fld_* independent field realizations and report the mean and standard deviation of performance metrics across these realizations.

### 5.2. Sensor Deployment and Experimental Parameters

We evaluate multiple scenarios of sensor counts, *N* ∈ {10, 40}, to investigate scaling behavior and the impact of spatial redundancy. We consider two distinct deployment policies: random and clustered. In random deployment, sensors are distributed to cover the field evenly with minimal clustering, thereby reducing local redundancy. On the other hand, the clustered deployment concentrates sensors in specific regions, intentionally introducing high redundancy within clusters while leaving other areas sparsely covered.

We use two representative budget levels for each sensor count in order to contrast sparse reporting and higher budget regimes in the main experiments while keeping the main comparison concise. Specifically, we set *B* ∈ {6, 25} for *N* = 10 and *B* ∈ {30, 100} for *N* = 40. Since *b_i_* = 0 is allowed, the low budget cases correspond to sparse reporting regimes in which the allocation rule jointly determines sensor activation and bit-depth assignment. In contrast, when the average number of bits per sensor is sufficiently larger than one, most sensors remain active, and the problem behaves more like conventional bit-depth allocation.

To examine the effect of budget selection, we additionally report two complementary analyses for *N* = 10. Appendix A presents the detailed high bit rate case (*B* = 50) (in this paper, the term “high bit” or “high budget” refers to the relatively larger bit budget cases in the main experiments, whereas “high bit rate” refers specifically to the additional experiments (with *N* = 10, *B* = 50) described in Appendix A.), corresponding to 5 bits per sensor on average. Appendix C provides a budget sensitivity analysis over *B* ∈ {6, 12, 25, 50}. The main text focuses on the representative budget settings.

For the stratified approximation, we scale *M* in accordance with the sensor count *N*: specifically, *M* ∈ {10, 30} for *N* = 10 and *M* = 1800 for *N* = 40. Determining *M* for each sensor count *N* is done through preliminary experiments to ensure sufficient approximation quality. Since computing the exact Shapley value is intractable, especially for larger *N* (e.g., *N* = 20, 40), we assess the approximation quality by comparing the sum of the approximate Shapley values, Σ*_i_Ø_i_*, with the grand coalitional utility *v*(*S*) that is obtained directly via

(29) $$v\left(S\right)=I\left(X_{S};X_{\bar{S}}\right).$$

We evaluate our Shapley allocation (both exact and approximate) and conduct a pilot stage to estimate stratum variances required for the Neyman approach. We draw 20 samples per stratum to obtain these estimates.

### 5.3. Reconstruction Procedure

Given a field realization **x***_u_* and sensor observation **x***_S_*, each method yields a bit-allocation vector **b**. We then reconstruct the full field using the GP posterior mean as defined in (11):

(30) $$\mu_{U}\left(b\right)=K_{US}{\left(K_{SS}+\Sigma_{q}\left(b\right)\right)}^{-1}X_{S}.$$

This reconstruction serves as the basis for RMSE-based metrics. In contrast, covariance-only metrics (MI and weighted posterior trace) are computed directly from **K** and **b** since they are independent of specific field realizations.

### 5.4. Evaluation Metrics

We give the five complementary metrics that capture both information-theoretic utility and reconstruction quality. The five metrics are not aggregated into a single score since they represent different design objectives. MI evaluates information content under quantization. Global RMSE measures average reconstruction accuracy over unattended points. B(Boundary)-RMSE focuses on regions far from sensors and is therefore sensitive to coverage holes. Worst-10% RMSE captures tail reconstruction errors; therefore, it evaluates reconstruction accuracy especially for the regions that exhibit higher measurement errors. WA-trace measures residual posterior uncertainty from the covariance model. Discrepancy among these metrics is treated as an important empirical finding rather than as an inconsistency.

#### 5.4.1. MI Under Quantization

For a bit allocation **b**, we compute an MI-based utility that accounts for both spatial correlation and quantization noise. This metric is used to measure how much information the quantized sensor observations provide about the target field. Using the sensor covariance **K***_SS_* and the diagonal quantization noise covariance **Σ***_q_*(**b**), the metric is evaluated as

(31) $$\mathrm{MI}\left(b\right)≜\frac{1}{2}\log_{2}\det\left(I+{K_{SS}\Sigma_{q}\left(b\right)}^{-1}\right).$$

Since **Σ***_q_*(**b**) decreases exponentially with bits, this metric explicitly captures the effect of bit allocation and spatial redundancy through **K***_SS_*.

#### 5.4.2. Global Reconstruction RMSE on Unattended Points

To focus on interpolation quality rather than trivial agreement at sensor grid locations, we evaluate RMSE over non-sensor grid points. This metric is consistent with the definition of *v*(*S*) and is defined as

(32) $$\mathrm{RMSE}\left(b\right)≜\sqrt{\frac{1}{\left|U\backslash S\right|}\sum_{u\in U\backslash S}{\left(x_{u}-\mu_{u}\left(b\right)\right)}^{2}}.$$

Here *μ_u_*(**b**) is the *u*-th component of the posterior mean ***μ****_U_*(**b**) given in (30). Consequently, the metric depends on **b** through both active sensors, that is, *S*_+_(**b**) = {*i* ∈ *S*; *b_i_* > 0}, and quantization noise covariance **Σ***_q_*(**b**).

#### 5.4.3. Boundary RMSE (B-RMSE)

We define a boundary subset *Θ* ⊂ *U* consisting of the top *p*% grid points farthest from their nearest sensors (we set *p* = 10 in our experiments). Then B-RMSE is defined as

(33) $$B-\mathrm{RMSE}\left(b\right)≜\sqrt{\frac{1}{\left|\Theta \right|}\sum_{u\in \Theta }{\left(x_{u}-\mu_{u}\left(b\right)\right)}^{2}}.$$

This metric is included since boundary regions often have fewer neighboring observations and can be more difficult to reconstruct accurately.

#### 5.4.4. Worst-10% RMSE (W10)

To quantify tail error, we compute the 90th percentile of absolute error over the entire grid:

(34) $$W10\left(b\right)≜{\mathrm{Quantile}}_{0.9}\left(\left\{\left|x_{u}-\mu_{u}\left(b\right)\right|\right\}\right).$$

This metric captures robustness against poorly reconstructed regions that may be hidden by the global RMSE.

#### 5.4.5. Weighted Posterior Trace (WA-Trace)

As a fifth metric, we evaluate a bit-dependent, covariance-only surrogate of reconstruction uncertainty based on the GP posterior covariance [29]. This metric quantifies how effectively a bit allocation **b** reduces posterior uncertainty across the domain without requiring field realizations, allowing for optional emphasis on regions of interest (ROIs).

Under the additive quantization-noise model, the posterior covariance for the active sensor set *S*_+_(**b**) is given by (12). Let *w* ∈ **R**^|*U*|^ be a nonnegative weight vector such that Σ*_u_*_∈*U*_*w_u_* = 1. The weighted average posterior trace (WA-trace) is defined as

(35) $$\mathrm{WA}-\mathrm{trace}\left(b\right)≜\sum_{u\in U}w_{u}{\left[K_{U|\tilde{S}}\left(b\right)\right]}_{uu}.$$

Equivalently, this presents a weighted average of posterior variances. Smaller values indicate lower residual uncertainty and superior reconstruction performance in expectation. In practice, we compute this efficiently as

(36) $$\mathrm{WA}-\mathrm{trace}\left(b\right)=\sum_{u\in U}w_{u}{\left[K_{UU}\right]}_{uu}-\sum_{u\in U}w_{u}{diag\left(K_{US_{+}}{\left(K_{S_{+}S_{+}}+\Sigma_{q}\left(b\right)\right)}^{-1}K_{S_{+}U}\right)}_{u}.$$

We set *w* to prioritize challenging regions. Specifically, we assign weights proportional to the prior variance, normalized to sum to one.

(37) $$w_{u}\propto {\left[K_{UU}\right]}_{uu},\;\sum_{u}w_{u}=1.$$

Similarly to MI(**b**), WA-trace(**b**) depends only on the covariance model and the bit allocation, allowing it to be computed once per experimental condition.

## 6. Experimental Results

This section presents the comparative performance of the five strategies. The results should be interpreted from a regime-dependent perspective. In particular, we examine whether contribution-aware allocation, geometry-aware allocation, information-driven allocation, or variance-based allocation is more suitable under each combination of deployment geometry, bit budget, and performance metric.

### 6.1. Experimental Protocol and Parameters

This section details the experimental procedures and parameters used throughout the evaluation, some of which are listed in Table 2.

With the parameters given in Table 1, we compare Shapley allocation (exactly one when *N* = 10, approximately one otherwise) against four heuristic baselines: uniform, Voronoi, greedy, and CVWA. To ensure a fair comparison, a consistent rounding rule is applied across all methods to produce the final integer allocations.

The sample budget for stratified sampling, *M*, is determined empirically by selecting the smallest *M* that approximately keeps the relative approximation error between the exact grand coalitional payoff and the sum of the approximate Shapley values within a 1% threshold. Concretely, we leverage the Shapley value’s efficiency axiom [27] as a calibration target,

(38) $$\sum_{i\in S}\emptyset_{i}=v\left(S\right),$$

and the relative approximation error is evaluated as

(39) $$\varepsilon_{eff}=\frac{\left|\sum_{i}{\hat{\emptyset }}_{i}-v\left(S\right)\right|}{\left|v\left(S\right)\right|}.$$

### 6.2. Approximation Quality and Execution Time

**Figure 1:**
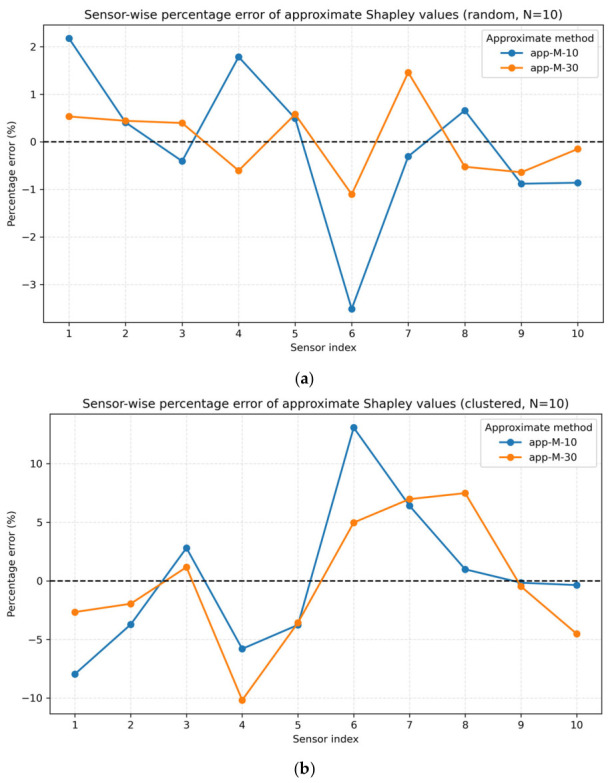
Sensor-wise percentage error of approximate Shapley values for *N* = 10: (**a**) random deployment; (**b**) clustered deployment.

The computation of exact Shapley values requires evaluating marginal contributions over an exponentially large set of coalitions. Therefore, exact Shapley values can be computed for small-scale cases. In this study, we use exact Shapley values only for the case of *N* = 10 to directly validate the accuracy of the approximate Shapley values at the individual sensor level. For the case of *N* = 40, we evaluate bit-allocation stability under different sampling budgets (*M*). We also evaluate approximate quality using efficiency-consistency, which measures how closely the sum of the estimated values matches the total utility ($\sum_{i}{\hat{\emptyset }}_{i}\approx v\left(S\right)$). Furthermore, we evaluate runtime scalability. The corresponding results are summarized in Figure 1 and Table 3.

#### 6.2.1. Approximation Quality via Sensor-Wise Error (Only for N = 10)

First, we measure the sensor-wise percentage error between the exact and approximate Shapley values for *N* = 10 under both random and clustered deployments, as shown in Figure 1. The percentage error for sensor *i* is defined as

(40) $${p\_error}_{i}=100\times \frac{{\hat{\emptyset }}_{i}-\emptyset_{i}}{\emptyset_{i}}.$$

It is observed that the approximation errors at sensors 6 and 7 are 1.1% and 1.5%, respectively, while they remain below 1% for the remaining sensors with *M* = 30 under the random deployment. In the clustered deployment, the approximation errors are slightly larger (up to approximately 10% at sensor 4) since the spatial redundancy and sensor heterogeneity are more pronounced. However, for *N* = 10, the approximation errors yielded with *M* = 30 do not exert a significant impact on the final integer bit-allocation results.

#### 6.2.2. Bit-Allocation Stability (Only for N = 40)

For *N* = 40, exact Shapley values are computationally infeasible, and therefore the exact impact of approximation error on the final bit-allocation results cannot be directly measured. Consequently, direct sensor-wise comparison is not possible within a reasonable time, and the bit-allocation vector obtained by exact Shapley values may not be identical to the one obtained by approximate values.

Previous studies have already demonstrated that the Neyman method provides an effective approach for approximating Shapley values while significantly reducing computational cost [6,7,30]. Nonetheless, we evaluate the approximation quality indirectly by examining whether the approximation error has a significant impact on the final bit-allocation decisions. To this end, we assess the stability of the resulting bit-allocation vectors obtained from different sampling budgets as

(41) $$D_{b}=\frac{1}{2B}\sum_{i=1}^{N}\left|b_{i}^{{(M}_{1})}-b_{i}^{\left(M_{2}\right)}\right|$$

where $b_{i}^{{(M}_{n})}$ denotes the number of bits allocated to sensor *i* with sampling budget *M_n_*. This metric represents the reallocation fraction of the total bit budget that is reassigned when the sampling budget changes.

At *N* = 40 and *B* = 100, we observe that the random deployment yields a reallocation fraction of 0.06 when increasing *M* from 1800 to 3000, which corresponds to 12 bits being reassigned. The sensor-wise allocation match ratio is 70%, indicating that 28 out of 40 sensors received the same bit allocation under the two sampling budgets. For the clustered deployment, the reallocation fraction is 0.04, corresponding to eight bits being reassigned. The sensor-wise allocation match ratio is 80%, indicating 32 out of 40 sensors received identical bit allocations.

These results show that increasing the sampling budget from 1800 to 3000 causes only limited changes in the final integer bit-allocation vectors, which implies the final allocation decision is relatively stable with respect to the sampling budget.

We also notice that this analysis does not provide the exact approximation error with respect to the true Shapley values for *N* = 40. Rather, it evaluates the practical stability of the final bit allocation under increased sampling effort.

#### 6.2.3. Approximation Quality via Efficiency Consistency

We use the efficiency-consistency evaluation as another scalable validation criterion. Key observations regarding the approximation quality are listed as follows:

- High efficiency: In most settings, the approximation is highly efficient, with *ε_eff_* falling well below 0.1%.
- Impact of *M*: Increasing the sample budget *M* generally improves efficiency; for instance, in the *N* = 10 clustered case, *ε_eff_* decreases from 0.52% at *M* = 10 to 0.066% at *M* = 30.

#### 6.2.4. Execution Time and Scalability

Execution time is dominated by repeated coalition-value evaluations. Accordingly, Table 2 reports the wall-clock time for each approximation setting; as previously mentioned, the runtime of computing the exact Shapley values is provided only for *N* = 10, as it becomes computationally infeasible to obtain exact values within a reasonable timeframe for larger *N*. Key observations are as follows:

- For *N* = 10, the approximation achieves a two-to-three order of magnitude speedup over the exact Shapley value computation, reducing the runtime from approximately 3900 s to just 7~21 s.
- For *N* = 40, the computation time for the approximate Shapley values scales to approximately 1000~1100 s.
- Notably, the exact computation time doubles whenever adding a single sensor: at *N* = 10, it requires roughly 3900 s, while at *N* = 11 and *N* = 12, the runtime grows to approximately 7600 and 17,600 s, respectively.

However, the computation time for the approximation still discourages real-world deployment. Therefore, the current approximation work should be interpreted as feasible for moderate-sized offline pre-computation scenarios. For very large-scale WSNs, additional structure should be exploited. Possible directions include decomposing the field into local regions, computing Owen values instead of Shapley values with restricting coalitions to spatially neighboring sensors, using Sparse GP approximations to reduce covariance computation cost, and parallelizing marginal contribution evaluations.

### 6.3. Downstream Reconstruction Performances

#### 6.3.1. Illustrations of Sensor Deployments

**Figure 2:**
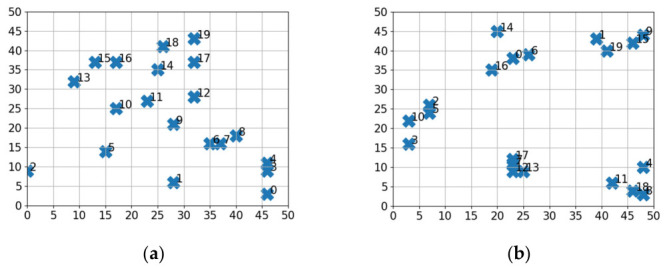
Sensor deployments for *N* = 20 on a 50 × 50 grid. The number annotated to each sesnor indicates sensor identification number: (**a**) random deployment; (**b**) clustered deployment with 5 clusters.

First, we illustrate the two deployment regimes used in our experiments. Figure 2 visualizes example sensor fields of 20 sensor locations on the 50 × 50 discretized field (we use the sensor fields of *N* = 20 only for illustrating the two deployment methods). The random deployment aims to achieve homogenous coverage across the entire field, reducing local redundancy and yielding relatively balanced interpolation distances. In contrast, the clustered deployment places sensors into five clusters, intentionally inducing high spatial redundancy among nearby sensors while leaving larger gaps between clusters. This clustered geometry amplifies the impact of allocation decisions under a limited bit budget: allocating many bits to sensors within a dense cluster may provide diminishing returns, whereas allocating more bits to sensors that improve homogeneous coverage can substantially reduce boundary and tail reconstruction errors.

#### 6.3.2. Performance Comparisons at *N* = 10

We present a comprehensive performance comparison for *N* = 10, where the exact Shapley value (SV) is computed. We also allow the approximate Shapley value (app-*xx*-M) to be directly benchmarked against both the Shapley value and heuristic baselines. In the figures of measurement results, app-10-M and app-30-M denote approximate Shapley allocation variants computed with sampling budgets *M* = 10 and *M* = 30, respectively.

**Figure 3:**
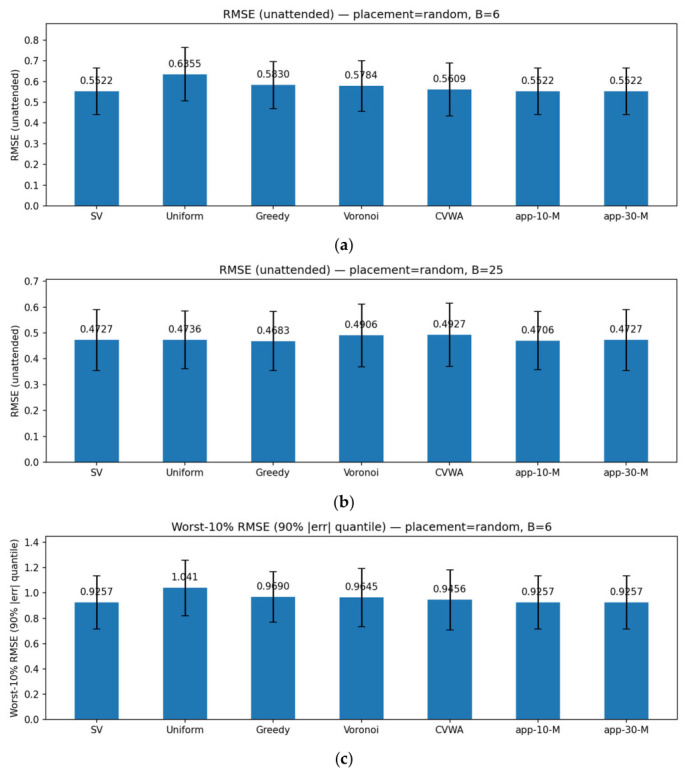

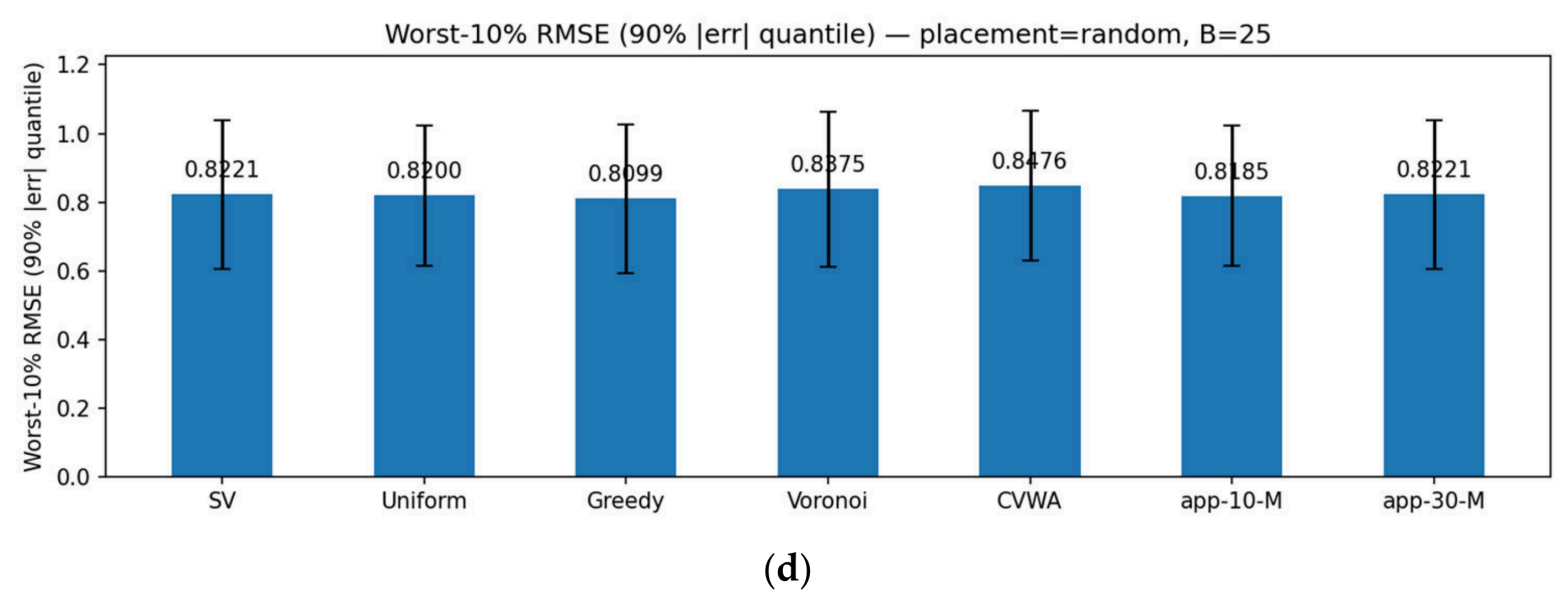
Mean of RMSEs and W10 measured in the sample fields with random deployment and *N* = 10: (**a**) RMSE measured with *B* = 6; (**b**) RMSE measured with *B* = 25; (**c**) W10 measured with *B* = 6; (**d**) W10 measured with *B* = 25.

We present representative results—specifically, RMSE and W10—using bar plots in Figure 2 and Figure 3. These plots show the mean values with standard-deviation error bars, computed over 20 independent field realizations (we have determined the number of field realizations by progressively increasing it until the reported mean and standard deviation estimates are stabilized). Therefore, when the mean values of two methods are close and their standard-deviation error bars substantially overlap, the difference is interpreted as marginal rather than as a decisive performance advantage.

Figure 3 shows bar plots of RMSE and W10 evaluated in random deployment. At *B* = 6, SV achieves the best performance across all three reconstruction metrics: RMSE, B-RMSE, and W10. Notably, app-10-M and app-30-M match SV results exactly (yielding identical mean and standard deviation), indicating that the approximation yields the same bit allocation and downstream behavior in this regime. Conversely, uniform allocation performs substantially worse (e.g., RMSE = 0.635, W10 = 1.041), confirming that contribution—or structure-aware allocation is beneficial under tight budgets, even in random deployments.

At *B* = 25, the performance of all methods largely converges with RMSE values within a narrow range (≈0.468~0.493). Greedy allocation attains the lowest RMSE (0.468) and W10 (0.81), while SV and app-30-M yield competitive results (RMSE = 0.473, W10 = 0.822). This convergence at high budget is expected as more sensors become active and quantization noise is reduced across the network.

**Figure 4:**
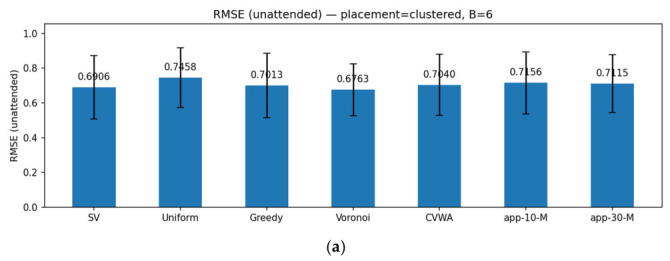

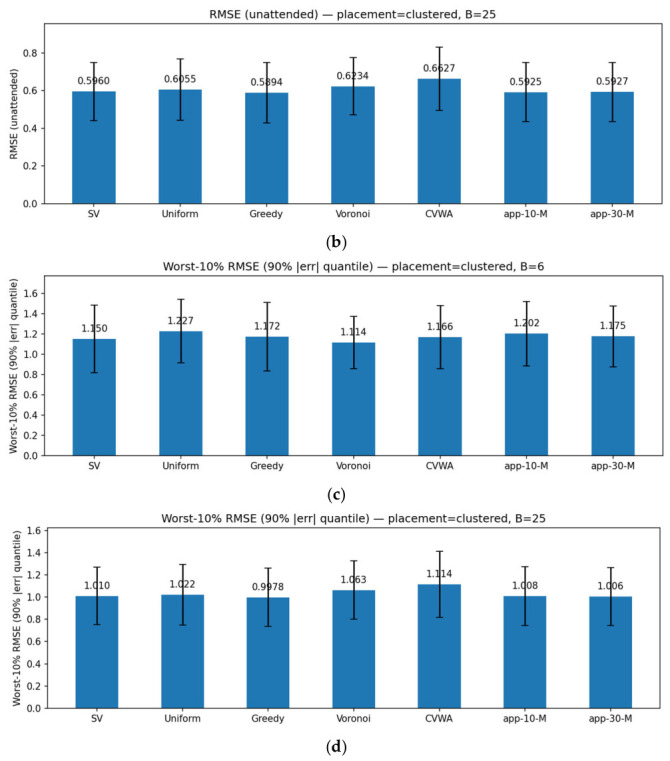
Mean of RMSEs and W10 measured in the sample fields of clustered deployment and *N* = 10: (**a**) RMSE measured with *B* = 6; (**b**) RMSE measured with *B* = 25; (**c**) W10 measured with *B* = 6; (**d**) W10 measured with *B* = 25.

Figure 4 shows the measured results with clustered deployment. In this environment, overall error levels are higher than in the random case as reflecting the inherent redundancy and limited field coverage. Interestingly, the relative performance of the methods is measured differently from the random deployment. At *B* = 6, Voronoi method performs best (RMSE = 0.676, B-RMSE = 0.890, W10 = 1.114) and outperforms SV (RMSE = 0.691, W10 = 1.150). This suggests that, in a clustered layout with tight budgets, a geometry-aware coverage proxy can be very effective by giving more bits to sensors that cover sparsely covered regions. At *B* = 25, greedy allocation achieves the best overall performance on RMSE and W10 (RMSE = 0.589, W10 = 0.998), and app-30-M yields the superior B-RMSE (0.883). SV remains highly competitive, showing similar results (RMSE = 0.596, W10 = 1.01). CVWA performs noticeably worse in this clustered deployment under this high budget (RMSE = 0.663, W10 = 1.114) and can be sensitive to the specific redundancy patterns.

In summary, the results exhibit that SV (and app-30-M) yields consistent performance gains in the low-budget and random-deployment regime. However, these differences diminish with a high budget, where all schemes converge towards similar reconstruction quality. In high-redundancy environments, Voronoi and greedy allocations show particularly strong performance, while SV and app-30-M remain near the top. This result indicates that contribution-aware allocation is robust; however, geometry- and information-driven heuristics can be competitive depending on the deployment structure and budget regime.

In addition to reporting the mean and standard deviation over independent field realizations, we perform statistical significance tests for the reconstruction error metrics. Since all allocation methods are evaluated using the same field realizations, the comparisons are paired across realizations. Hereby, we perform a paired *t*-test to test the paired differences in each metric. Further details are described in Appendix B.

#### 6.3.3. Performance Comparisons at *N* = 40

**Figure 5:**
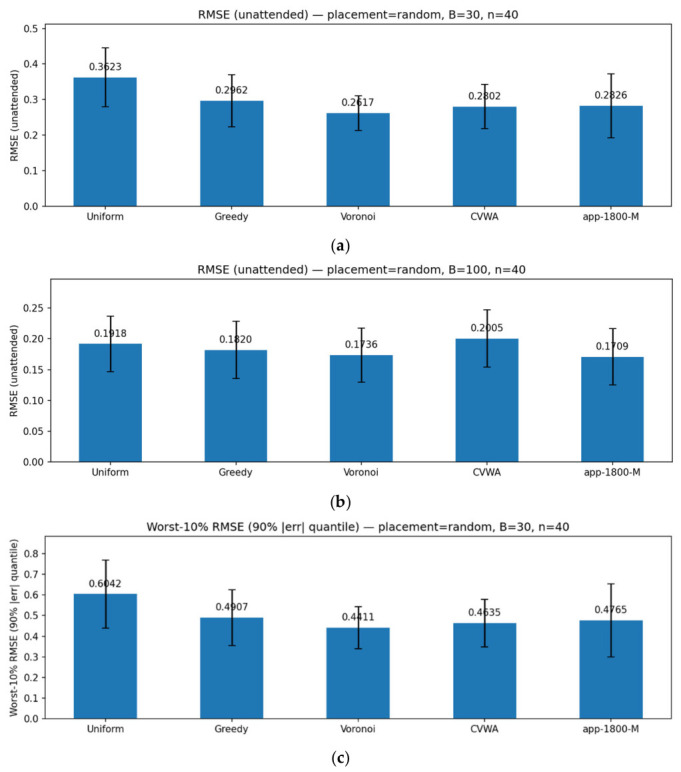

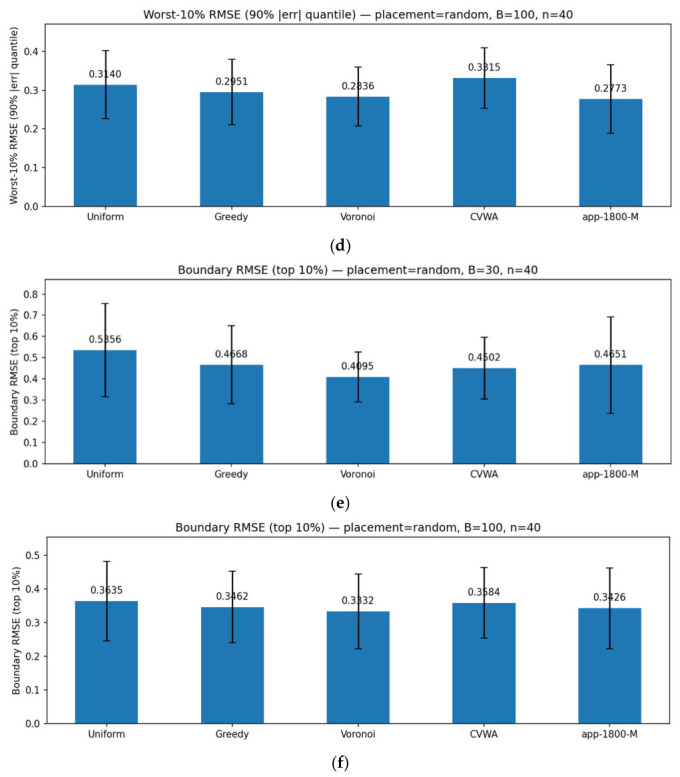
Mean of RMSEs, W10, and B-RMSEs measured in the sample fields of random deployment and *N* = 40: (**a**) RMSE measured with *B* = 30; (**b**) RMSE measured with *B* = 100; (**c**) W10 measured with *B* = 30; (**d**) W10 measured with *B* = 100; (**e**) B-RMSE measured with *B* = 30; (**f**) B-RMSE measured with *B* = 100.

**Figure 6:**
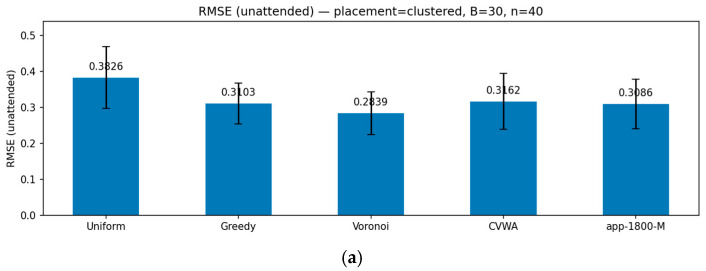

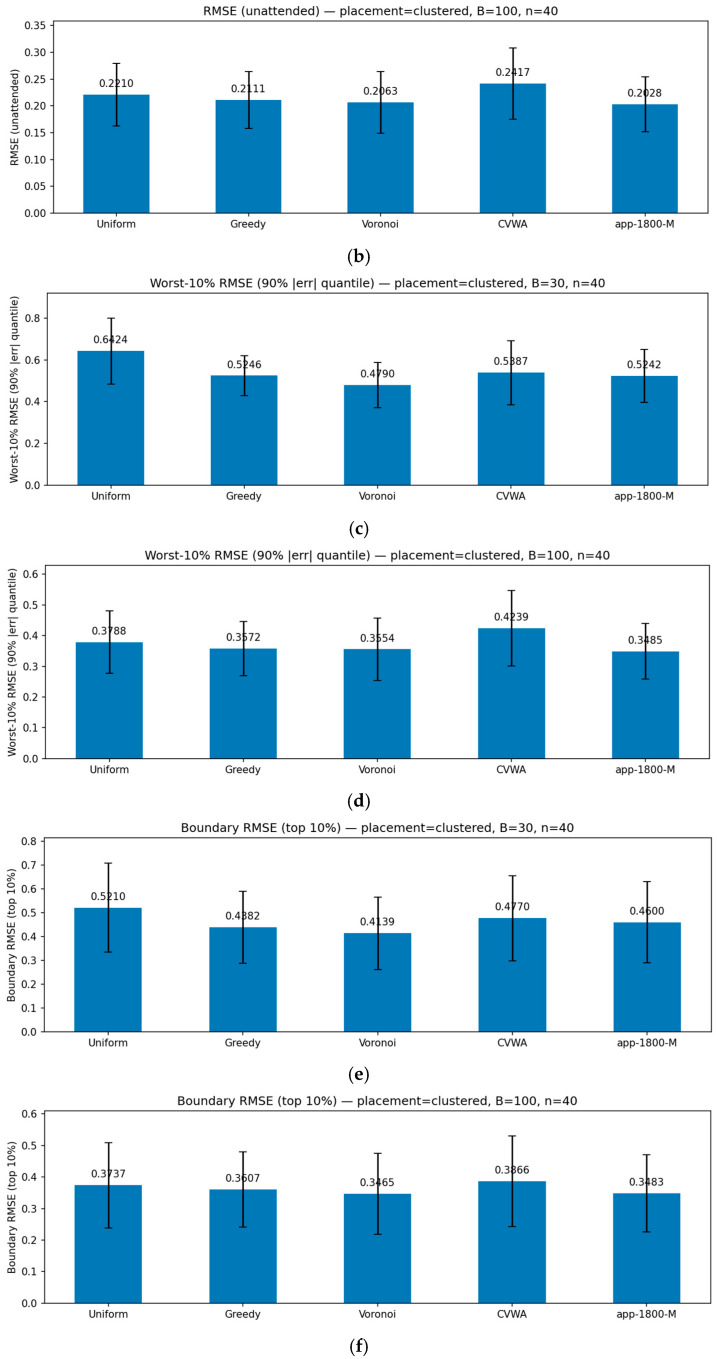
Mean of RMSEs, W10, and B-RMSEs measured in the sample fields of clustered deployment and *N* = 40: (**a**) RMSE measured with *B* = 30; (**b**) RMSE measured with *B* = 100; (**c**) W10 measured with *B* = 30; (**d**) W10 measured with *B* = 100; (**e**) B-RMSE measured with *B* = 30; (**f**) B-RMSE measured with *B* = 100.

We now examine the case of *N* = 40, which represents a relevantly large scale where the computation of the exact Shapley value is no longer feasible. We therefore employ bit allocations derived from approximate Shapley values using *M* = 1800—selected via the efficiency-consistency criterion described in Section 6.2.1. Consistent with *N* = 10, we consider two representative bit budgets: a tight budget (*B* = 30) and a high budget (*B* = 100). The results of random deployment and clustered deployment are presented in Figure 5 and Figure 6, respectively.

Figure 5 shows the results evaluated under random deployment. At *B* = 30, Voronoi allocation outperforms others across all reconstruction metrics, indicating geometry-aware coverage can be highly effective in low-budget regimes. CVWA ranks second, slightly outperforming Shapley allocation. This observation can be explained by the following two factors:

- First, CVWA is inherently more aligned with reconstruction-error-based metrics since it directly targets local predictive uncertainty. Especially when the budget is constrained and only a limited subset of sensors can be activated with sufficient resolution, prioritizing sensors that maximize conditional novelty often brings reduced reconstruction errors.
- Second, the use of integer budgets and the allowance for sensor silencing mean that even minor shifts in bit placement can change which sensors are activated. These discretization effects may lead to slight ranking reversals among methods.

At *B* = 100, overall errors decrease, and the performance of the various methods partially converges. Shapley allocation achieves the lowest RMSE and W10 (RMSE = 0.171, W10 = 0.277). While Voronoi allocation yields the lowest B-RMSE (0.333), Shapley remains highly competitive with a slightly lower value (0.343). The key trend is that Shapley allocation becomes increasingly favorable as the budget grows, reinforcing the idea that contribution-aware allocation provides stable, global improvements once a sufficient number of sensors are activated.

Figure 6 plots the results measured with clustered deployment. With a low budget (*B* = 30), Voronoi allocation provides the best overall performance (RMSE = 0.284, W10 = 0.479, B-RMSE = 0.414), while Shapley allocation remains competitive (RMSE = 0.309, W10 = 0.524). In this highly redundant and resource-constrained scenario, the geometry-aware approach excels by minimizing redundant bit spending within dense clusters and prioritizing coverage in sparse regions, which is the objective that Voronoi cell size effectively captures. At high budget (*B* = 100), Shapley allocation achieves the lowest RMSE (=0.203) and W10 (=0.349). While Voronoi allocation remains best for B-RMSE (=0.347), it is almost tied with Shapley allocation (=0.348). This indicates that even with higher budgets, the clustered geometry preserves a benefit for allocations that explicitly address coverage gaps, especially on boundary-focused error.

In summary, Voronoi allocation shows the lowest reconstruction error under tight budget constraints regardless of the sensor deployment. In contrast, Shapley allocation yields the best (or near-best) results at high budgets. Furthermore, it is noticed that boundary error (B-RMSE) is most responsive to the geometry-aware allocation approach.

### 6.4. Covariance-Only Metrics

In this section, we report two covariance-only metrics—MI and WA-trace—that depend only on the covariance model and the bit-allocation vector. These metrics are computed once per experimental condition without any field realizations. They provide complementary perspectives by quantifying (i) the information content of the quantized sensor reports and (ii) the residual posterior uncertainty across the field.

#### 6.4.1. Performance Comparisons at *N* = 10

We first examine *N* = 10 under both deployments and budgets *B* ∈ {6, 25}.

**Figure 7:**
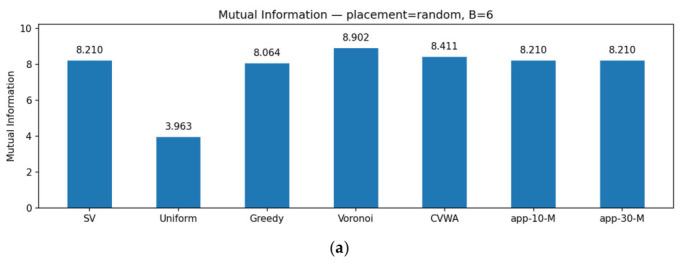

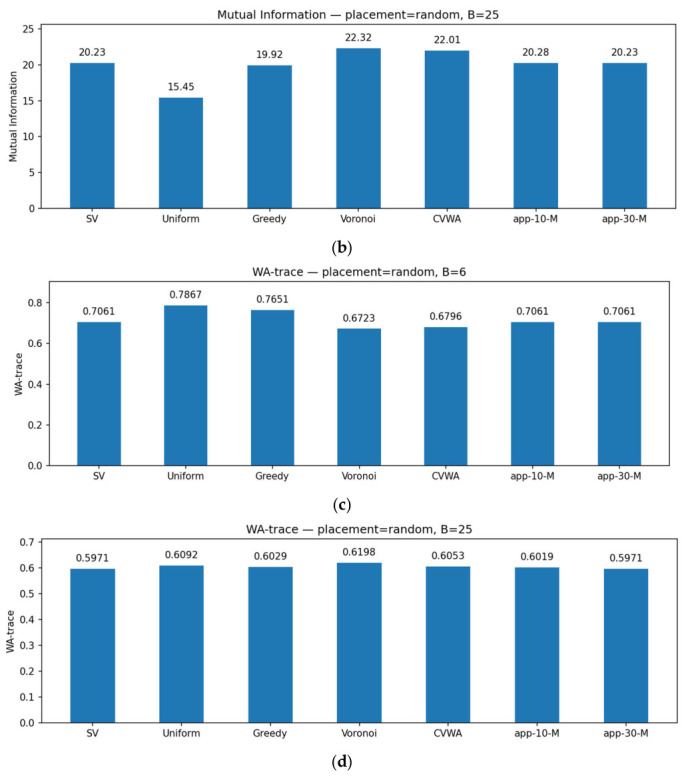
MIs and WA-traces measured in the sample fields of random deployment and *N* = 10: (**a**) MI measured with *B* = 6; (**b**) MI measured with *B* = 25; (**c**) WA-trace measured with *B* = 6; (**d**) WA-trace measured with *B* = 25.

Figure 7 shows bar plots of MIs and WA-traces measured with random deployment. At *B* = 6, Voronoi allocation achieves the highest MI (8.90) and also the lowest WA-trace (0.672), followed closely by CVWA (MI: 8.41, WA-trace: 0.68) and SV (MI: 8.21, WA-trace: 0.706). This indicates that under tight budgets in random deployment, the geometry-aware allocation shows the best performance in both metrics. At *B* = 25, Voronoi allocation again yields the highest MI (22.32), with CVWA following at 22.01. However, WA-trace is reduced by SV and app-30-M (0.597). As the budget grows, MI and WA-trace continue to capture diverging aspects of the allocation outcome, highlighting the distinction between increasing information gain and diminishing residual uncertainty.

**Figure 8:**
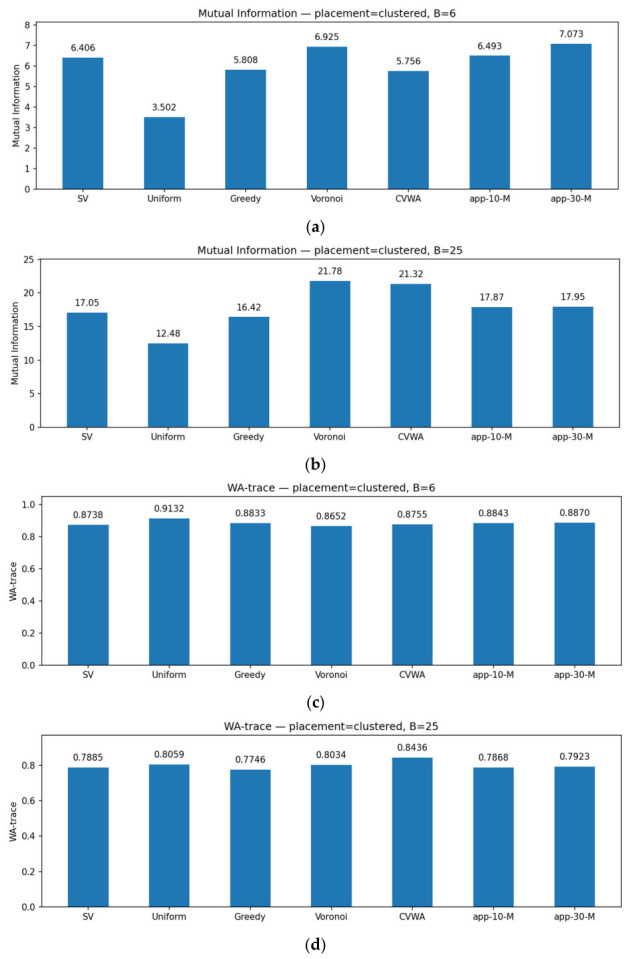
MIs and WA-traces measured in the sample fields of clustered deployment and *N* = 10: (**a**) MI measured with *B* = 6; (**b**) MI measured with *B* = 25; (**c**) WA-trace measured with *B* = 6; (**d**) WA-trace measured with *B* = 25.

Figure 8 presents the bar plots for MI and WA-trace measured under clustered deployment. At *B* = 6, the highest MI is achieved by app-30-M (7.07), with Voronoi allocation ranking second (6.93). In contrast, Voronoi allocation yields the minimum WA-trace (0.865), slightly outperforming SV (0.874). These results indicate that in a clustered geometry under tight budgets, Voronoi allocation is most effective at reducing global posterior uncertainty (WA-trace), whereas app-30-M maximizes the information gain (MI). At *B* = 25, Voronoi allocation yields the highest MI (21.78), while greedy allocation achieves the minimum WA-trace (0.775), with SV performing comparably (0.789). This suggests that in high-budget clustered cases, greedy allocation can be the most effective at reducing posterior uncertainty within the weighted ROI even if it does not strictly maximize the information gain (MI).

Across both deployment scenarios, MI tends to be maximized by Voronoi allocation or CVWA, while WA-trace is often minimized by SV (in random deployment) or Voronoi/greedy allocation (in clustered deployment), depending on budget. These results indicate that while MI and WA-trace are both informative, they are not interchangeable: MI captures the information content within the quantized sensor vector, whereas WA-trace directly targets residual posterior uncertainty across the field.

#### 6.4.2. Performance Comparisons at *N* = 40

We next present the results measured at *N* = 40. The budget applied here is *B* ∈ {30, 100}.

**Figure 9:**
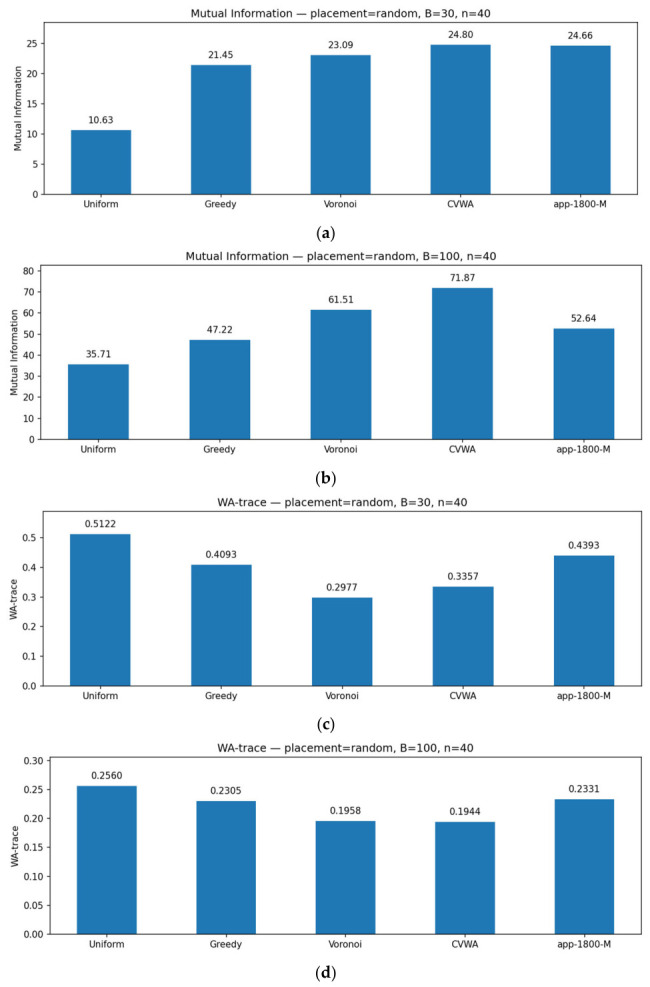
MIs and WA-traces measured in the sample fields of random deployment and *N* = 40: (**a**) MI measured with *B* = 30; (**b**) MI measured with *B* = 100; (**c**) WA-trace measured with *B* = 30; (**d**) WA-trace measured with *B* = 100.

Figure 9 plots the results measured with random deployment. At *B* = 30, MI is maximized by CVWA (24.80) and remains high with SV (24.66). In contrast, WA-trace is minimized by Voronoi allocation (0.298), which outperforms CVWA (0.336) and SV (0.439). At *B* = 100, CVWA again maximizes MI (71.87) and yields the minimum WA-trace (0.194), with Voronoi allocation following very closely (0.196). In this scenario, SV shows substantially lower MI and higher WA-trace. These results suggest that CVWA consistently delivers the best performance in MI regardless of bit budgets in random deployment.

**Figure 10:**
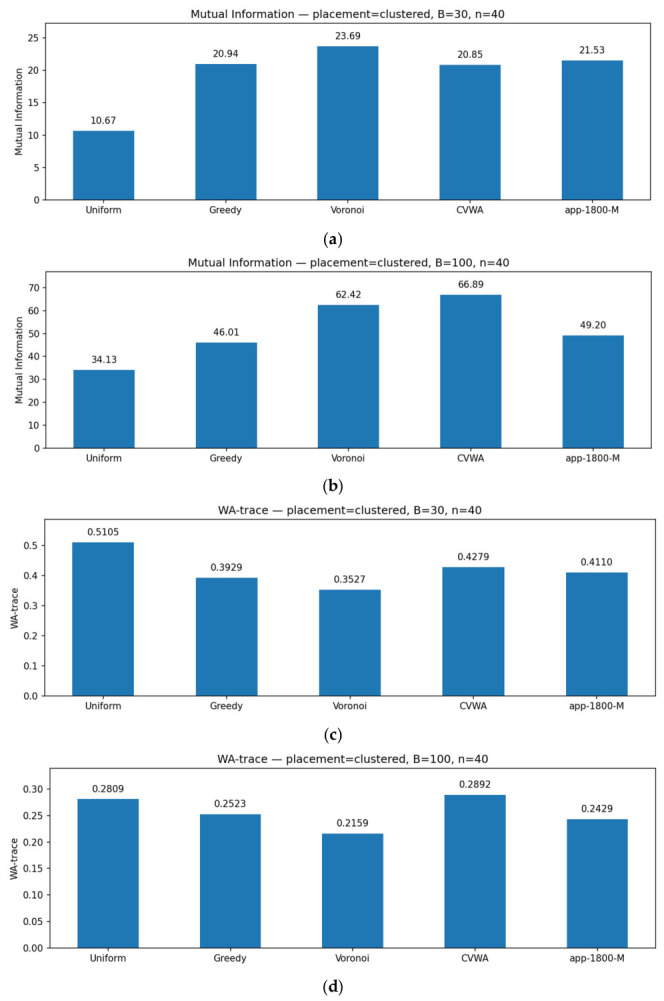
MIs and WA-traces measured in the sample fields of clustered deployment and *N* = 40: (**a**) MI measured with *B* = 30; (**b**) MI measured with *B* = 100; (**c**) WA-trace measured *B* = 30; (**d**) WA-trace measured with *B* = 100.

Figure 10 shows the results measured with clustered deployment. At *B* = 30, Voronoi allocation shows the best performance in MI (23.69) and WA-trace (0.353), while SV performs lower on both (MI: 21.53, WA-trace: 0.411). At *B* = 100, MI is maximized by CVWA (66.89), followed by Voronoi allocation (62.42). In contrast, Voronoi allocation maintains minimum WA-trace (0.216), while uniform allocation shows intermediate performance (0.281). These results demonstrate that Voronoi allocation consistently delivers robust performance across both metrics and budget regimes in clustered environments.

In conclusion, MI performance increasingly favors CVWA, especially at a large scale and under high budgets, while WA-trace strongly favors Voronoi allocation in clustered regimes. These results demonstrate that maximizing MI does not necessarily minimize ROI-weighted posterior uncertainty. This discrepancy is particularly evident in clustered deployment, where coverage gaps dominate the reconstruction performance.

### 6.5. Summary of Experimental Results

We summarize the performance of each allocation method in Table 4 and Table 5, identifying the experimental configurations (*N*, *B*, deployment) and the metric where each method achieves the “Best” or “2nd Best” performance results. In these tables, “SV” indicates allocation via exact Shapley values and “App.” denotes allocation via approximate ones. This representation highlights that methodological superiority is highly context-dependent: a specific method may be optimal for certain metrics in one regime, yet underperform in others.

The summary shows that the best allocation strategy is strongly regime- and metric-dependent. In tight budget settings, geometry-aware allocation often performs well because spatial coverage becomes critical when sensor silencing occurs, although contribution-aware allocation is best for *N* = 10, *B* = 6 with random deployment. This behavior is more pronounced in clustered deployments since sensors within the same cluster tend to provide redundant information, whereas regions outside the clusters remain sparsely covered. Therefore, spatial coverage has a stronger effect on reconstruction quality in clustered deployments than in random deployments, where sensor locations already provide more diverse spatial information. In higher-budget settings, Shapley allocation and its approximation become competitive for global and tail reconstruction errors, particularly for *N* = 40, *B* = 100, but Voronoi or CVWA can still be preferred for boundary error or covariance-based metrics. Therefore, the results should not be interpreted as a universal dominance of one method. Instead, they indicate that contribution-aware, geometry-aware, information-driven, and variance-based allocation principles are suitable for different deployment geometries, budget regimes, and evaluation metrics.

We notice that “SV” and “App” belong to the same Shapley allocation family, but they are reported separately since approximation errors and integer bit mapping can lead to different final allocation vectors. Therefore, when “App.” Achieves the best performance for a specific metric, the result should be interpreted as the performance of the approximate Shapley-based allocation under that regime, not as a claim that the approximation is theoretically superior to the exact Shapley value.

The standard-deviation error bars indicate that some performance gaps are small relative to the variability across field realizations. Therefore, the results should be interpreted mainly in terms of consistent trends across sensor deployments, bit budgets, and metrics, rather than as a strict total ordering of all methods in every individual case.

The Shapley allocation uses a bit-independent MI-based cooperative game to score sensor contributions before bit allocation, but it does not directly minimize RMSE, boundary RMSE, or worst-10% RMSE. After the scores are mapped to integer bits, reconstruction performance is affected by sensor silencing, rounding, quantization noise, deployment geometry, interpolation distance, and specific field realizations. Therefore, a method with high information-theoretic utility does not necessarily yield the lowest reconstruction error. This mismatch is one of the main empirical findings of this study and supports our metric-by-metric comparison rather than aggregation into a single score.

## 7. Concluding Remark

This paper studies per-reporting-round bit allocation under a global bit budget in spatially correlated sensor fields, where the number of bits assigned to each sensor determines its quantization quality, and consequently, its effective contribution to field reconstruction. We employ five allocation schemes: Shapley-value-based contribution-aware allocation, uniform allocation, Voronoi-based geometry-aware allocation, greedy MI-driven allocation, and conditional variance-based allocation under random and clustered sensor deployments, different bit budgets, and multiple evaluation metrics.

The experimental results highlight several key findings. First, the stratified random sampling reduces the computation time of Shapley values with high approximation quality. Second, the best allocation strategy is context-dependent: under a tight budget, geometry-aware (Voronoi) allocation frequently produces the lowest reconstruction errors, revealing the essence of spatial coverage under stringent quantization constraints and the existence of sensor silencing. In contrast, under high budgets, Shapley allocation becomes highly competitive and, in several scenarios, best for global and tail reconstruction errors. Moreover, we also observe that Shapley allocation yields the lowest reconstruction errors for *N* = 10 at tight bit budgets. Third, covariance-only metrics (MI and WA-trace) do not always align with reconstruction metrics, which underscores the necessity of evaluating allocation strategies with multiple complementary metrics rather than a single criterion.

From a practical viewpoint, the proposed comparative framework can guide reporting bit allocation in resource-constrained WSN applications. In smart city or environmental monitoring systems, sensors located in highly redundant regions may be assigned fewer bits, while sensors providing more distinctive information for field reconstruction may receive higher reporting resolution. In disaster-response or UAV-assisted sensing scenarios, where communication opportunities and bandwidth may vary over time, the per-reporting-round bit budget can be adapted to the available communication resources. The results of this study suggest that the choice of allocation strategy should depend on the operating regime: sparse-reporting regimes require careful sensor activation and bit assignment, whereas high-bit-rate regimes show smaller differences among allocation methods.

Future work is streamlined into two main directions. The first direction is to extend the modeling and objective functions. This includes non-stationary sensor fields, where spatial redundancy may vary across the sensing region, and alternative contribution criteria such as Fisher information or reconstruction-error-based utilities. The second direction is to improve practical scalability. This includes adaptive online bit allocation for time-varying communication constraints and localized or hierarchical implementations that can reduce the computational burden in large-scale WSNs. These extensions would allow the proposed framework to better support more realistic and larger sensor-network deployments.

## Figures and Tables

**Table 1:** Comparison with previous bit-rate and bit-allocation studies.

Study	Main Objective	Resource Constraint	Main Metrics	Redundancy-Awareness?
[10]	Distributed filtering in WSNs	Bit-rate constraint in communication link	Filtering performance/error dynamics	No
[13]	Bit allocation for object tracking	Fixed total bit budget	Tracking performance	No
[16]	Bit allocation for cooperative visual sensing in VANETs	Fixed total bit budget	Perception performance	No
This work	Contribution-aware bit allocation for field reconstruction	Per-reporting-round bit budget	RMSE/weighted posterior trace	Yes

**Table 2:** Experimental parameters.

Item	Settings	
Field grid size	50 × 50	
*α* in the covariance model given in (4)	0.3	
Sensor deployments	random/clustered	
Number of samples used for finding the optimal *m_k_*	20	
Bit budgets (*B*)	*N* = 10	6, 25
	*N* = 40	30, 100
Sample budgets for stratified sampling (*M*)	*N* = 10	10, 30
	*N* = 40	1800
Number of clusters for the clustered sensor deployments	*N* = 10	3
	*N* = 40	8

**Table 3:** Efficiency consistency and execution time of the approximation.

N	Deployment	*M*	Time (s)	$\sum_{i}{\hat{\emptyset }}_{i}$	*v*(*S*)	*ε*_*eff*_ (%)
10	random	10	8	229.996	230.071	0.0326
		30	21	230.155		0.0365
	clustered	10	7	208.061	206.987	0.5189
		30	19	206.850		0.0662
40	random	1800	1063	806.703	806.778	0.0093
	clustered	1800	1030	732.363	732.912	0.0749

**Table 4:** The best and 2nd best performing allocation methods in *N* = 10. Hyphen in “2nd best” field implies two methods share the top rank with identical results in “Best” category. “SV” and “App.” indicate exact Shapley value and approximation variants of Shapley value, respectively.

Deployment	*B*	Metric	Best	2nd Best
random	6	RMSE	SV, App.	-
		B-RMSE	SV, App.	-
		W10	SV, App.	-
		MI	Voronoi	CVWA
		WA-trace	Voronoi	CVWA
	25	RMSE	Greedy	App.
		B-RMSE	App.	Greedy
		W10	Greedy	App.
		MI	Voronoi	CVWA
		WA-trace	SV, App	-
clustered	6	RMSE	Voronoi	SV
		B-RMSE	Voronoi	Greedy
		W10	Voronoi	SV
		MI	App.	Voronoi
		WA-trace	Voronoi	SV
	25	RMSE	Greedy	App.
		B-RMSE	App.	Greedy
		W10	Greedy	App.
		MI	Voronoi	CVWA
		WA-trace	Greedy	App.

**Table 5:** The best- and 2nd-best-performing allocation methods in *N* = 40.

Deployment	*B*	Metric	Best	2nd Best
random	30	RMSE	Voronoi	CVWA
		B-RMSE	Voronoi	CVWA
		W10	Voronoi	CVWA
		MI	CVWA	App.
		WA-trace	Voronoi	CVWA
	100	RMSE	App.	Voronoi
		B-RMSE	Voronoi	CVWA
		W10	App.	Voronoi
		MI	CVWA	Voronoi
		WA-trace	CVWA	Voronoi
clustered	30	RMSE	Voronoi	App.
		B-RMSE	Voronoi	Greedy
		W10	Voronoi	App.
		MI	Voronoi	CVWA
		WA-trace	Voronoi	Greedy
	100	RMSE	App.	Voronoi
		B-RMSE	Voronoi	App.
		W10	App.	Voronoi
		MI	CVWA	Voronoi
		WA-trace	Voronoi	App.

**Figure A1:**
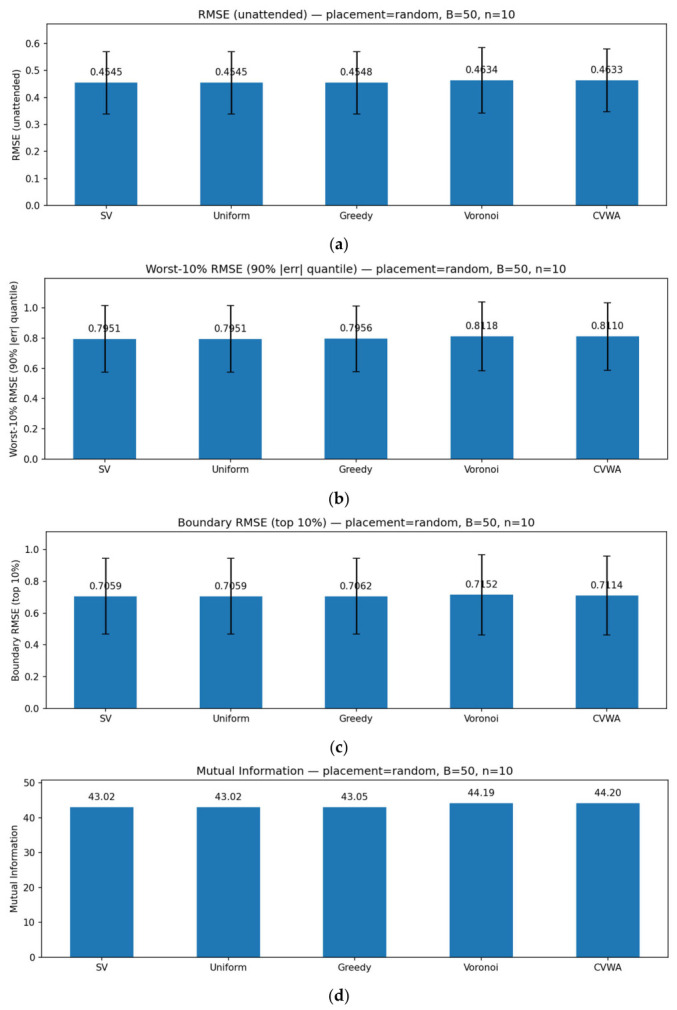

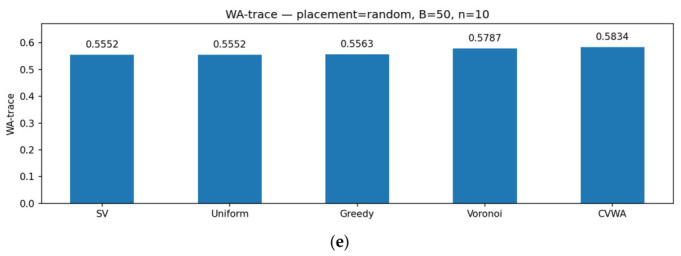
Performance comparison in the sample fields of random deployment with *N* = 10 and *B* = 50: (**a**) global reconstruction error measured by RMSE; (**b**) tail error measured by worst-10% RMSE; (**c**) boundary reconstruction error measured by B-RMSE; (**d**) bit-dependent mutual information; (**e**) weighted posterior uncertainty measured by WA-trace.

**Figure A2:**
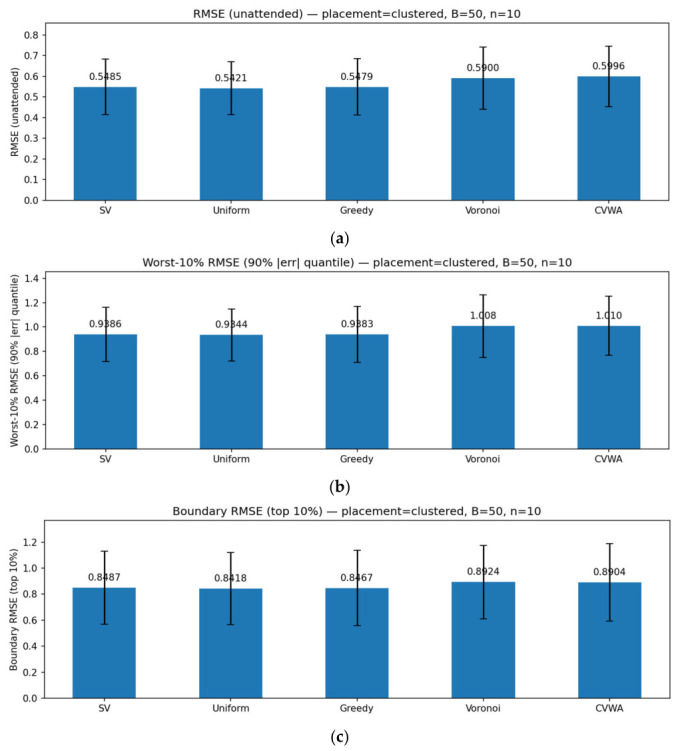

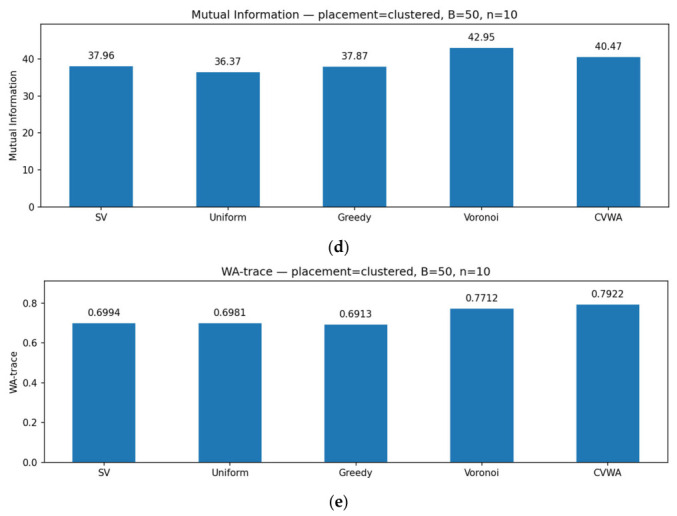
Performance comparison in the sample fields of clustered deployment with *N* = 10 and *B* = 50: (**a**) global reconstruction error measured by RMSE; (**b**) tail error measured by worst-10% RMSE; (**c**) boundary reconstruction error measured by B-RMSE; (**d**) bit-dependent mutual information; (**e**) weighted posterior uncertainty measured by WA-trace.

**Figure A3:**
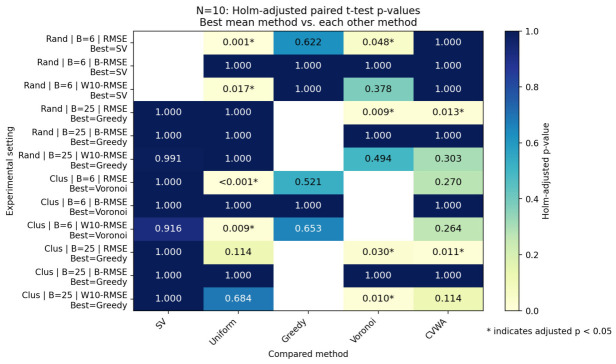
Holm-adjusted paired *t*-test *p*-values for *N* = 10. Each row (denoted “Experimental setting” corresponds to one experimental setting, and each cell (denoted “Compared method”) reports the adjusted *p*-value for the comparison between the method yielding the best mean value and another allocation method. “Rand” and “Clus” denote random deployment and clustered deployment, respectively, and “Best = Voronoi” implies Voronoi allocation yields the best mean value in the given setting. The methods “app-10-M” and “app-30-M” are excluded for better readability. An asterisk indicates *p* < 0.05.

**Figure A4:**
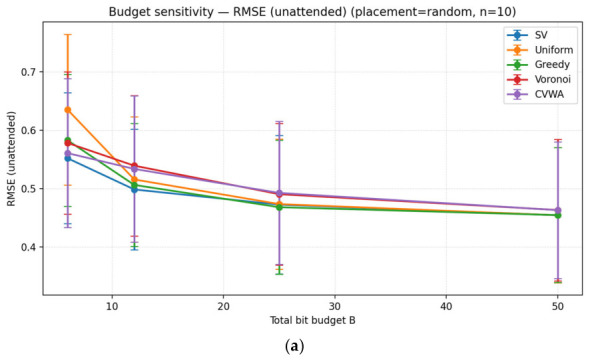

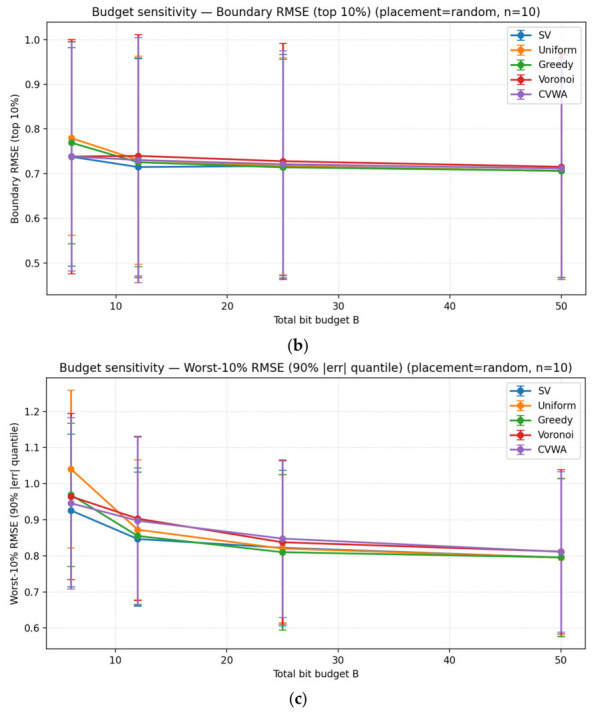
Budget sensitivity analysis for *N* = 10 under random deployment. Exact Shapley allocation—denoted SV—and heuristics are compared across *B* ∈ {6, 12, 25, 50} in terms of the reconstruction error metrics: (**a**) RMSE; (**b**) B-RMSE; (**c**) W10.

**Figure A5:**
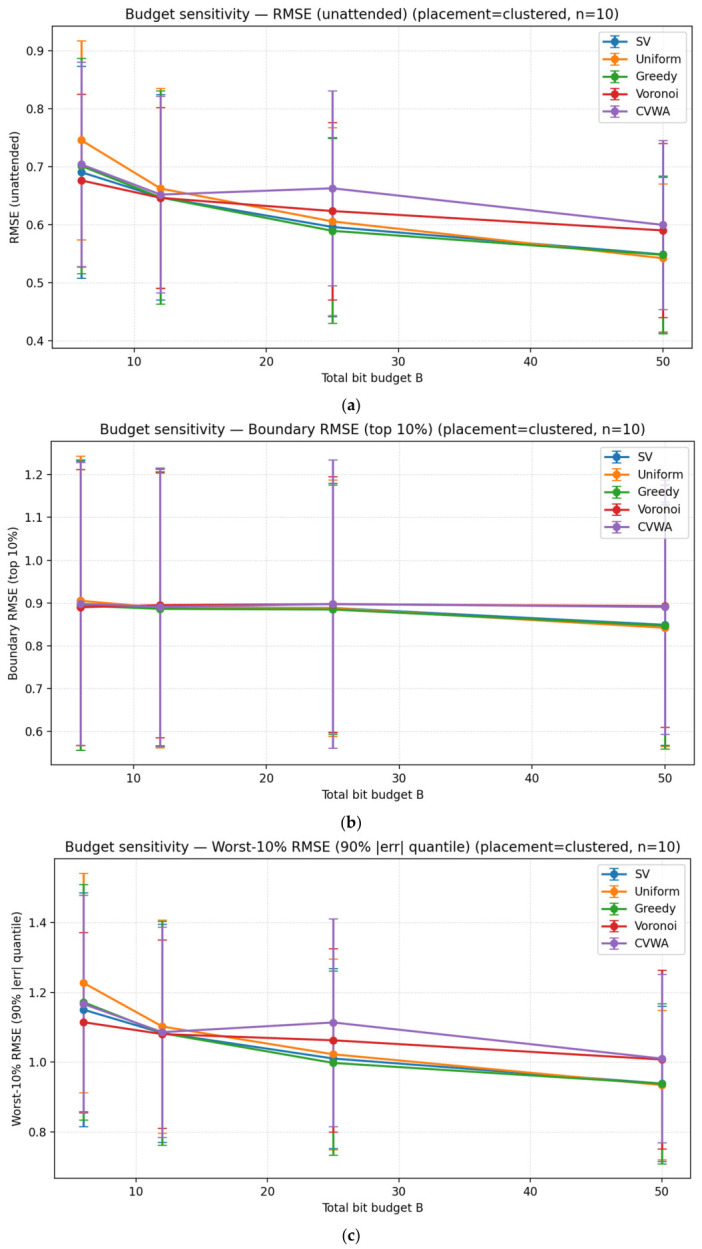
Budget sensitivity analysis for *N* = 10 under clustered deployment. Exact Shapley-allocation—denoted SV—and heuristics are compared across *B* ∈ {6, 12, 25, 50} in terms of the reconstruction error metrics: (**a**) RMSE; (**b**) B-RMSE; (**c**) W10.

## Data Availability

The data supporting the findings of this study are available from the corresponding author upon request.

## Appendix A. Additional Evaluation with a High Bit Rate

This appendix reports an additional high-bit-rate experiment for *N* = 10 and *B* = 50, corresponding to five bits per sensor on average. This setting reduces the effect of sensor silencing and allows us to examine the behavior of the allocation methods when bit allocation becomes more dominant than sensor activation. We compare SV (allocation with exact Shapley values) with the other heuristic baselines.

Figure A1 and Figure A2 plot the results measured with random deployment and clustered deployment, respectively. In this setting, we observe that the reconstruction error metrics are very close across the methods regardless of deployments. This occurs since, in the high-bit-rate regime, all the allocation methods produce identical or nearly identical bit-allocation vectors. As a result, the benefit of nonuniform allocation becomes less dominant. CVWA and Voronoi produce slightly higher reconstruction errors, although the difference remains small compared with those observed in low-budget settings.

We also observe that CVWA and Voronoi achieve the highest MI, whereas SV, uniform, and greedy show slightly lower MI in random deployment. However, the lowest WA-trace is yielded by SV and uniform, followed closely by greedy. This indicates that maximizing MI does not necessarily lead to the lowest reconstruction error metrics or weighted posterior uncertainty in this setting. These results also show that different performance criteria can rank allocation methods differently.

For the clustered deployment, uniform allocation yields the lowest global RMSE, boundary RMSE, and worst-10 error, while greedy and SV produce very similar performance. Voronoi and CVWA show higher reconstruction errors. With regard to MI, Voronoi achieves the largest value, followed by CVWA, whereas uniform gives the lowest MI. However, the WA-trace result shows a different tendency: greedy achieves the lowest WA-trace, followed by uniform and SV. Therefore, even in the high-bit-rate regime, the best-performing method depends on the metric used for evaluation.

Overall, the results with high bit rate regime show that higher bit budgets reduce the performance gap among allocation methods. In this regime, most allocation methods assign the same or very similar numbers of bits to sensors, often approaching uniform allocation. As a result, the reconstruction error metrics become nearly indistinguishable across methods. In contrast, MI and WA-trace are still dependent on allocation principles. These results support the main conclusion that no single allocation method is uniformly best across all criteria, deployments, and budget regimes. The impact of the allocation strategy is most visible in low- and high-budget regimes, whereas method-dependent reconstruction differences diminish in the high-bit-rate setting.

## Appendix B. Statistical Significance Analysis

This appendix provides additional statistical significance results for the reconstruction error metrics: RMSE, B-RMSE, and W10. Pairwise paired *t*-tests are performed since all allocation methods are evaluated on the same set of independent field realizations. The resulting *p*-values are adjusted using the Holm correction to account for multiple pairwise comparisons. A difference is considered statistically significant when the Holm-adjusted *p*-value is smaller than 0.05 [31].

### Appendix B.1. Paired Test Results for N = 10

Figure A3 summarizes the paired test results for *N* = 10. Each row corresponds to one experimental setting, including the deployment type, bit budget, and reconstruction error metric. For each setting, the method with the best mean performance is compared with the other allocation methods. The cell values represent Holm-adjusted *p*-values, and an asterisk indicates statistical significance at the 0.05 level. We omit the approximation variants “app-10-M” and “app-30-M” from this figure for better clarity.

As shown in this figure, statistically significant differences are mainly observed in some low-budget cases. In particular, when the bit budget is tight, the best mean method often shows statistically significant improvement over weaker baselines. However, in several high-bit-budget cases, many pairwise differences are not statistically significant after Holm correction. This indicates that small mean differences should be interpreted as marginal rather than as decisive superiority.

### Appendix B.2. Paired Test Results for N = 40

In the low-budget case *B* = 30, Voronoi-based allocation achieves the lowest mean reconstruction error for RMSE, boundary RMSE, and worst-10% RMSE in both random and clustered deployments. The paired tests show that this advantage is statistically significant in several comparisons, especially for RMSE. In the clustered deployment, Voronoi is significantly better than all other compared methods for RMSE. For boundary RMSE and worst-10% RMSE, however, the significance is not universal across all method pairs. Thus, the low-budget result supports the usefulness of geometry-aware allocation under severe communication constraints, but it should still be interpreted in a metric-dependent manner.

For the moderate-budget case *B* = 100, the best mean performance is obtained by SV depending on the deployment and metric. However, the corrected paired-test results show that not all mean differences are statistically significant. In particular, boundary RMSE differences are often not significant after Holm correction, while RMSE and worst-10% RMSE show significant differences mainly against weaker-performing baselines such as Uniform and CVWA. This indicates that the apparent ranking among the stronger methods should be interpreted cautiously.

For the high-bit-rate case *B* = 200, which corresponds to five bits per sensor on average, the differences among allocation methods become less pronounced. Uniform allocation often gives the lowest mean RMSE or worst-10% RMSE, while greedy gives the lowest mean boundary RMSE in the clustered deployment. Nevertheless, the statistically significant differences are mainly observed against CVWA or other clearly weaker cases, and several comparisons among the stronger methods are not significant. This result is consistent with the interpretation that, when the bit budget is sufficiently large, different allocation rules tend to produce similar reconstruction performance.

Overall, the *N* = 40 paired-test results support the main conclusion that no single allocation method is uniformly superior across all settings. Geometry-aware allocation is statistically supported in several low-budget cases, SV is competitive in moderate-budget settings, and performance differences diminish in the high-bit-rate regime.

## Appendix C. Bit Budget Sensitivity Analysis

This appendix presents an additional budget-sensitivity analysis for *N* = 10. The purpose of this analysis is to examine whether the trends observed in the main experiments are robust across a broader range of bit budgets. Since exact Shapley value computation is feasible for *N* = 10, this analysis uses the exact Shapley allocation—denoted SV in the figures—and the heuristic baselines only: uniform, greedy, Voronoi, and CVWA. Approximation variants of Shapley values are excluded in this analysis.

We evaluate *B* ∈ {6,12,25,50}, corresponding to 0.6, 1.2, 2.5, and 5.0 bits per sensor on average. Thus, the evaluated budgets cover sparse reporting, intermediate budget, and high-bit-rate regimes. The reconstruction error metrics are RMSE, boundary RMSE, and worst-10% RMSE, and the results are reported with standard deviation error bars over independent field realizations in Figure A4 and Figure A5.

The sensitivity results are consistent with the representative results summarized in Table 4. For the random deployment, SV shows the lowest or nearly lowest reconstruction error across the evaluated budgets. At *B* = 6, SV achieves the lowest RMSE, boundary RMSE, and worst-10% RMSE among the compared methods. At *B* = 12, SV again gives the best reconstruction error values. At *B* = 25, greedy slightly outperforms SV in the reconstruction error metrics, but SV remains close to greedy and uniform. At *B* = 50, SV and uniform yield identical reconstruction-error values, and greedy produces almost the same performance. This indicates that, in the random deployment, contribution-aware allocation is effective under low and intermediate budgets, while the difference among methods becomes very small when the bit budget is sufficiently large.

For the clustered deployment, Voronoi performs best under tight budgets, particularly at *B* = 6, where spatial coverage is critical since several sensors may receive zero bits. At *B* = 12, Voronoi remains competitive for RMSE and worst-10% RMSE, while greedy gives the lowest boundary RMSE. At *B* = 25, greedy becomes the best method for the reconstruction error metrics when approximate SV are excluded. At *B* = 50, uniform, greedy and SV produce similar results, while Voronoi and CVWA are less effective. These observations confirm that the preferred allocation strategy depends on the budget regime, deployment geometry, and performance metric rather than following a universal ranking.
